# Nuclear factor interleukin 3 and metabolic dysfunction-associated fatty liver disease development

**DOI:** 10.1038/s42003-024-06565-z

**Published:** 2024-07-24

**Authors:** Yung-Ni Lin, Jia-Rou Hsu, Chih-Lin Wang, Yi-Chen Huang, Jzy-Yu Wang, Chun-Ying Wu, Li-Ling Wu

**Affiliations:** 1 Department and Institute of Physiology, College of Medicine, National Yang Ming Chiao Tung University, Taipei, Taiwan; 2 Institute of Clinical Medicine, National Yang Ming Chiao Tung University, Taipei, Taiwan; 3 Department of Family Medicine, National Cheng Kung University Hospital, Tainan, Taiwan; 4 Institute of Biomedical Informatics, National Yang Ming Chiao Tung University, Taipei, Taiwan; 5 Health Innovation Center, National Yang Ming Chiao Tung University, Taipei, Taiwan; 6 Microbiota Research Center, National Yang Ming Chiao Tung University, Taipei, Taiwan; 7 Division of Translational Research, Taipei Veterans General Hospital, Taipei, Taiwan; 8 Institute of Public Health, College of Medicine, National Yang Ming Chiao Tung University, Taipei, Taiwan; 9 Department of Public Health, China Medical University, Taichung, Taiwan

**Keywords:** Non-alcoholic fatty liver disease, Microbiome

## Abstract

This study investigates sex-specific effects in a gain-of-function model to evaluate *Nfil3* function in relation to high-fat diet (HFD)-induced metabolic dysfunction-associated steatotic liver disease (MASLD) and gut microbiota (GM)-induced alterations in the bile acid (BA) profile. MASLD is induced in both wild type and *Nfil3*-deficient (NKO) C57BL/6 J mice through an HFD. The hepatic immune response is evaluated using flow cytometry, revealing that NKO mice exhibit lower body weight, serum triglyceride (TG) levels, tissue injury, inflammation, and fat accumulation. The *Nfil3* deletion reduces macrophage counts in fibrotic liver tissues, decreases proinflammatory gene and protein expression, and diminishes gut barrier function. Alpha and beta diversity analysis reveal increased GM alpha diversity across different sexes. The *Nfil3* gene deletion modifies the BA profile, suggesting that negative feedback through the *Nfil3-FXR-FGF15* axis facilitates BA recycling from the liver via enterohepatic circulation. Therefore, inhibiting *Nfil3* in the liver offers a viable treatment approach for MASLD.

## Abstract (web summary)

This study explores the role of nuclear factor interleukin 3 (*Nfil3*) in metabolic dysfunction-associated fatty liver disease (MASLD) development, revealing sex-specific effects and gut microbiota influence on bile acid metabolism.

## Introduction

Sex differences (SDs) are well established in metabolism and metabolic disorders, such as insulin sensitivity, body composition, energy balance, and metabolic disease incidence^1–4^. However, the underlying mechanisms of these SDs remain to be elucidated. The liver is continuously exposed to intestine-originated metabolites, as it receives a preponderance of blood supply from the gut. Therefore, gut dysbiosis plays a crucial role in metabolic disorder development^5–7^. However, whether gut microbiota (GM)-derived signaling contributes to SDs in metabolic disorders remains unclear. Hepatic and bacterial enzyme-produced bile acids (BAs) are essential for metabolism and immune response regulation^8–10^. Thus, the GM may modulate BAs and farnesoid X receptor (FXR) signaling, which affect host metabolism^5,11–14^ and immunity^11,15^.

A typical Western diet (high-fat and sugar) poses a considerable health risk in humans. As a model, a high-fat diet (HFD) induces obesity and steatosis in mice. In response to an HFD, the BA synthesis becomes dysregulated^16^. *Nfil3* expression is elevated in patients with liver cirrhosis and cancer and presents a possible therapeutic target^17^. However, studies on the role of *Nfil3* in metabolic dysfunction-associated steatotic liver disease (MASLD) are limited. Additionally, the role of *Nfil3* in regulating GM-mediated BA profile changes remains unclear. Our objective was to investigate the relationship between GM and BAs and the development of metabolic syndrome in patients with SDs. We proposed the hypothesis that variations in GM composition and the associated BA metabolites play a significant role in the progression of SDs in metabolic disorders where *Nfil3* is involved. In this study, we analyzed the associations between GM and BAs related to an HFD in male and female wild type and *Nfil3*-deficient (NKO) mice. Ultimately, our study findings will determine whether *Nfil3* expression regulation, which is linked to both sex and diseases, could be the key to preventing and treating metabolic disorders.

## Results

### *Nfil3* is upregulated, while *FXR* is downregulated in patients with clinically obesity

**Fig. 1:**
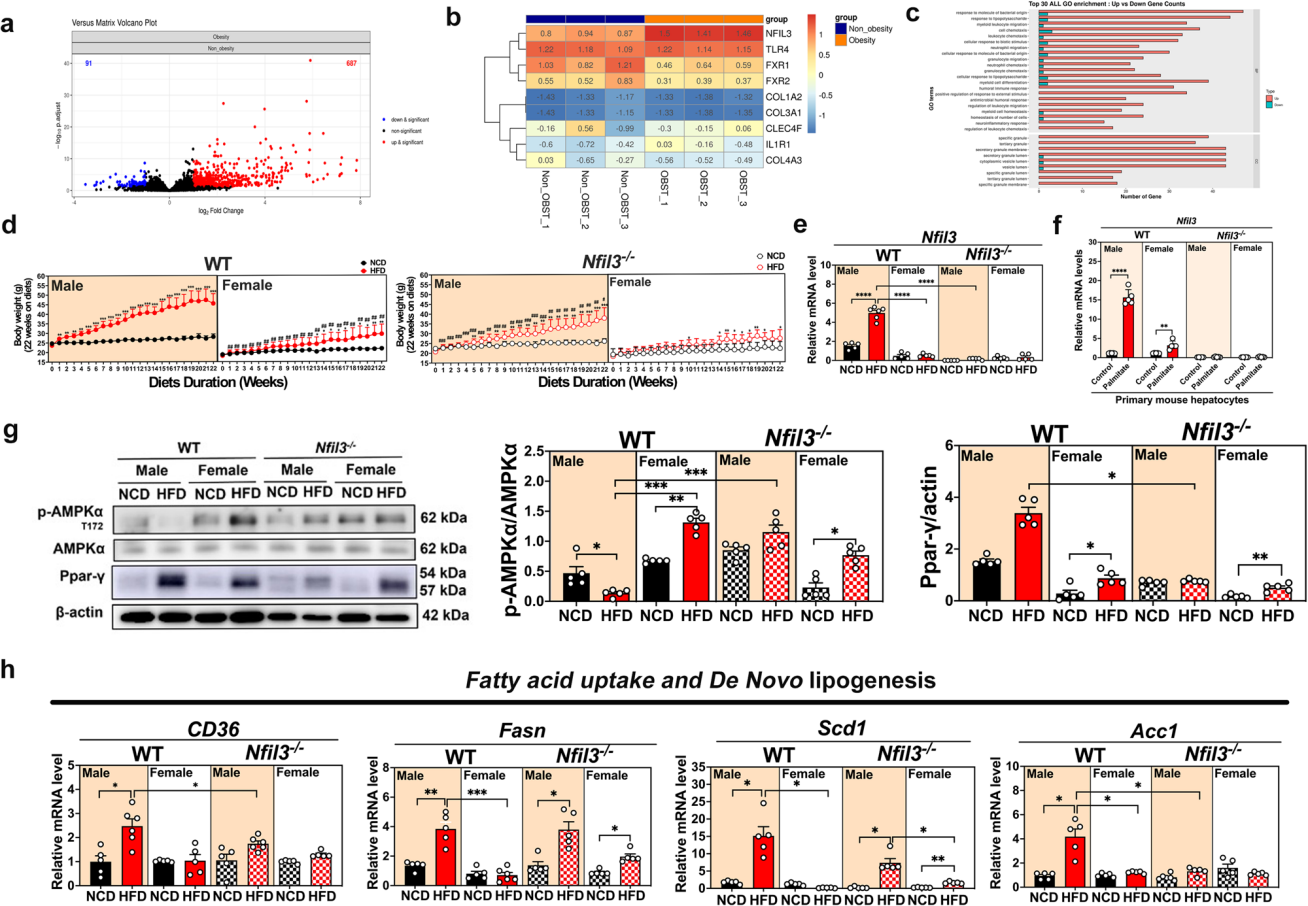
Human clinical data on non-obesity and obesity, and sex-specific de novo lipogenesis in WT and *Nfil3*-deficient (NKO) mice given a HFD. **a** The volcano plot analysis revealed the differences in peripheral blood mononuclear cell (PBMC) gene expression between patients with and without obesity. **b** PBMC heat map of gene expression differences between patients with and without obesity. **c** Enrichment of gene ontology (GO) analysis. **d** Male and female WT and NKO mice that were fed an HFD for 22 weeks exhibited weekly weight changes (y-axis: grams, x-axis: weeks). **e** Liver *Nfil3* gene expression in HFD-fed male and female WT and NKO mice. **f** Primary mouse hepatocytes were isolated from WT and NKO mice and then treated with palmitate (400 μM) for 24 h before harvest for mRNA analysis. The mRNA levels of *Nfil3* were examined by RT-qPCR. **g** Western blot analysis of liver lipid synthesis-related protein expression in WT and NKO mice fed an HFD. Phosphorylated AMPK protein expression analysis. Protein expression statistics for PPAR-r. **h** *Cd36, Fasn, Scd1*, and *Acc1* mRNA expression is compared between the livers of WT male and female mice and NKO mice fed an HFD. The format for presenting experimental data is the standard error of the mean (SEM). The following statistical differences are shown: *P < 0.05; **P < 0.01; ***P < 0.001; ****P < 0.0001.

A total of 687 genes were elevated in the peripheral blood mononuclear cells of patients with obesity (Fig. 1a). Notably, the expression of *Nfil3* and *TLR4* was significantly higher in obese patients compared to non-obese individuals, while *FXR* expression was markedly lower (Fig. 1b). RNA-seq data revealed that *Nfil3* is intricately linked to the innate *TLR4* response to intestinal bacteria and to negative regulatory genes influenced by BA, as shown through functional annotation and pathway assignment. Gene ontology (GO) term analysis was employed to categorize genes by similar functions and associations. Differentially expressed genes (DEGs) were predominantly grouped in various GO categories related to distinct biological processes, cellular components, and molecular functions. Figure 1c displays the top 30 GO categories for 21 different biological processes and 9 cellular components. Among these, the cellular component “cytoplasm” was most enriched, involving DEGs in the secretory granule membrane, as well as secretory granule and cytoplasmic vesicle lumens. For the 21 biological processes, the categories “response to molecules of bacterial origin,” “response to lipopolysaccharides,” and “myeloid leukocyte migration” were highly enriched. Consequently, NKO mice were used to further investigate these mechanisms.

### *Nfil3* deletion protects against HFD-induced MASLD

Under a continuous HFD for 22 weeks, male WT mice had increased body weight (BW) than female WT mice. In contrast, NKO-HFD mice exhibited diminished BWs (Fig. 1a and Supplementary Fig. S1a). This observation was consistent under equivalent caloric intake conditions (Supplementary Fig. S1b). This result indicated that NKO mice gained significantly less weight than WT mice. Although female NKO mice gained slightly less weight than female WT-HFD mice, the difference was not statistically significant (Fig. 1d and Supplementary Fig. S1a). Quantitative polymerase chain reaction analysis revealed that *Nfil3* expression was higher in male WT-HFD mice than in female WT-HFD mice. NKO mice exhibited reduced *Nfil3* expression in the liver compared with WT mice, demonstrating successful *Nfil3* gene deletion (Fig. 1e). To determine if the observed response is conserved in primary mouse hepatocytes, we conducted an in vitro challenge with palmitate on both WT and NKO mice. The treatment with palmitate caused a significant increase in *Nfil3* expression in male WT PMH cells relative to their female counterparts. In contrast, both male and female PMH from *NKO* mice exhibited considerably lower *Nfil3* levels than those from WT mice (Fig. 1f). Additionally, levels of low-density lipoprotein, high-density lipoprotein, triglycerides (TG), and cholesterol (CHO) were notably higher in male WT-HFD mice than in female WT-HFD mice. However, with the exception of a slight rise in CHO levels, all lipid profiles were markedly lower in NKO-HFD male mice than in WT-HFD male mice (Supplementary Fig. S2d). These findings underscore the role of *Nfil3* in blood lipid regulation, highlighting that its deletion leads to a reduction in lipid abnormalities.

After 4 weeks of being fed an HFD, male WT-HFD mice had significantly higher glucose levels than female WT-HFD, mice and male NKO-HFD mice had significantly lower fasting blood glucose levels than male WT-HFD mice (Supplementary Fig. S1c). After 20 weeks of HFD feeding, an oral glucose tolerance test (OGTT) was performed. Male WT-HFD mice reached their OGTT peak 15 min after glucose solution injection, whereas female WT-HFD mice peaked 30 min later (Supplementary Fig. S1d), indicating that male WT-HFD mice exhibited poor blood glucose control. The male and female NKO-HFD group OGTT values peaked at 15 min (Supplementary Fig. S1d). The area under the curve was greater in male NKO-HFD mice than in male WT-HFD mice, but the difference was not statistically significant (Supplementary Fig. S1e). The NKO and WT mice exhibited comparable glucose tolerance, whereas male WT-HFD mice exhibited impaired glucose metabolism.

After 22 weeks of being fed an HFD, male WT-HFD mice had a greater liver weight (LW) than male WT-normal chow diet (NCD) and female WT-HFD mice. In addition, female NKO-HFD mice had higher LW than female NKO-NCD mice (Supplementary Fig. S1d). Male WT-HFD mice also displayed significantly more white and brown adipose tissues than female WT-HFD mice (Supplementary Fig. S2a,b). Male WT-HFD mice exhibited more adipocyte-stained tissues than female WT-HFD mice. Male NKO-HFD mice possessed fewer adipocytes and adipose cells than male WT-HFD mice. In both WT-HFD and NKO-HFD female mice, the adipose cell size decreased (Supplementary Fig. S2c). Thus, HFD-induced adiposity regulated metabolic function via *Nfil3* deletion. The NKO mice gained less weight and had decreased glucose tolerance and fasting glucose levels. The NKO mice exhibited reduced fat cell size, fat content, organ weight, and organ morphology. Conclusively, *Nfil3* deletion may protect against obesity-related metabolic dysregulation.

### *Nfil3* deletion decreases mRNA levels of genes related to fatty acid uptake and de novo lipogenesis in the liver

To further explore the mechanism by which *Nfil3* regulates lipid accumulation after HFD feeding, fatty acid uptake and de novo fatty acid synthesis factors were examined. Western blotting was used to analyze the lipid synthesis-related proteins. Male WT-HFD mice exhibited higher PPAR-γ protein expression than male NKO-HFD mice (Fig. 1g). AMP-activated protein kinase (AMPK) phosphorylation was higher in female WT-HFD mice than in male WT-HFD or female WT-NCD mice. NKO mice exhibited similar results. Male NKO-HFD mice displayed greater AMPK phosphorylation than male WT-HFD mice, which improved the MASLD scores (Fig. 1g). Male WT-HFD mice expressed considerably higher levels of *Cd36, Fasn, Acc1*, and *Scd1* mRNA than male NKO-HFD mice (Fig. 1h). These results indicated that HFD-induced lipid accumulation in the liver was modulated by the effects of *Nfil3* on fatty acid uptake and de novo lipogenesis. *Nfil3* deletion prevents HFD-induced endogenous lipogenesis and delays MASLD development.

### *Nfil3* deletion protects mice of both sexes against HFD-induced liver damage and steatosis

**Fig. 2:**
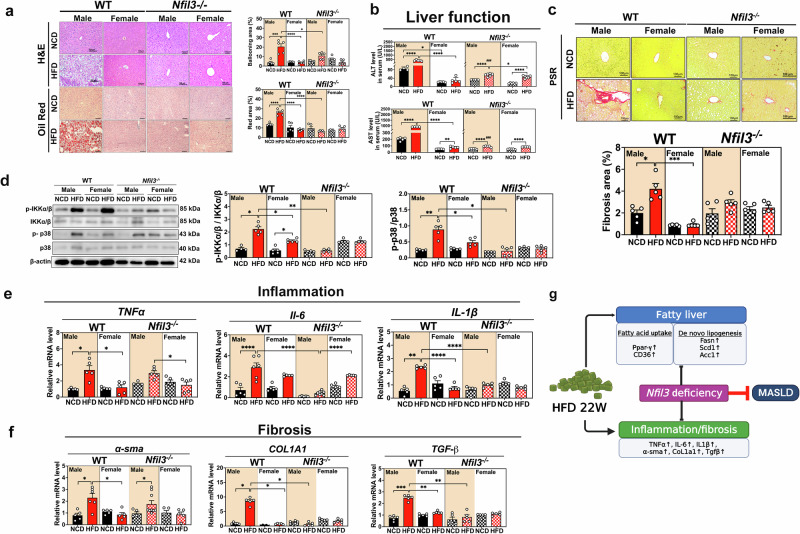
Male and female wild type (WT) and *Nfil3*-deficient (NKO) mice fed a HFD exhibited hepatic tissue injury and lipid droplet accumulation. **a** Liver tissue stained with hematoxylin and eosin (H&E) and Oil Red O to identify tissue injury and lipid droplet production. Scale = 100 μm, 100× magnification. Statistical analysis of the percentage of enlarged hepatic area. The statistical accumulation of lipids in the liver. Ballooning area (%). Oil red O area quantification. **b** Liver function in terms of ALT and AST levels. **c** Hepatic fibrosis staining with Picrosirius Red (PSR) in HFD male and female WT and NKO mice (Scale bar = 100 μm), 200× magnification. Fibrosis area (%). **d** Western blot of proteins related to hepatic inflammation in HFD-fed WT and NKO mice. Phosphorylation associated with liver inflammation and IB kinase/p38 protein expression analysis. **e** *Tnf-α, Il-6*, and *Il-1β* gene expression in the livers of male and female WT and NKO mice fed an HFD. **f** Hepatic fibrosis quantification in groups. *α-sma, Col1a1*, and *Tgf-β* gene expression in HFD male and female WT mice. Standard error of the mean (SEM) is used to present experimental data. **g** An illustration of the summary. The schematic diagram was created with BioRender.com. The following statistical differences are indicated: *P < 0.05; **P < 0.01; ***P < 0.001; ****P < 0.0001.

Compared with female WT-HFD mice, male WT-HFD mice exhibited liver tissue ballooning, unsaturated coloration, and liver tissues contained red lipid droplets. Male WT-HFD mice had more liver injury and steatosis than female WT and NKO mice (Fig. 2a). NKO-HFD mice exhibited significantly reduced liver function (Fig. 2b), indicating reduced liver injury. These results implied that *Nfil3* may play a role in the hepatic inflammatory cascade.

### *Nfil3* deletion protects mice of both sexes against HFD-induced liver fibrosis and inflammation

The IKK/p38 MAPK and p38 MAPK pathways were activated in male WT-HFD mice, increasing the levels of pro-inflammatory markers compared with those of male NKO-HFD mice (Fig. 2d). Female WT and NKO mice had lower hepatic mRNA expression of the proinflammatory markers *Tnf-α, Il-1β, Il-6*, and the chemokine, monocyte chemoattractant protein-1 (*Mcp-1*) compared with those of male WT-HFD mice (Fig. 2e and Supplementary Fig. S1g). The inflammatory protein expression was reduced in NKO mice, indicating reduced inflammation. Picrosirius red staining of liver tissues revealed that only male WT-HFD mice had increased fibrotic tissues. Male NKO-HFD mice displayed less liver fibrosis than male WT-HFD mice. Male WT-HFD mice showed significantly more liver fibrosis than male WT-NCD and NKO-NCD mice (Fig. 2c). Next, we examined whether the expression of fibrosis-related genes *α-sma, Col1a1*, and *Tgf-β* was lower in female WT-HFD mice than in male WT-HFD mice. Several fibrosis-related genes were substantially downregulated in the male and female NKO-HFD mice (Fig. 2f). These findings confirmed that male NKO-HFD mice exhibited significantly less hepatic fibrosis than male WT-HFD mice. *Nfil3* gene deletion decreased HFD-induced liver fibrosis and inflammation in male MASLD mice (Fig. 2g).

### *Nfil3* deletion protects mice of both sexes against HFD-induced liver immune microenvironment modulation

**Fig. 3:**
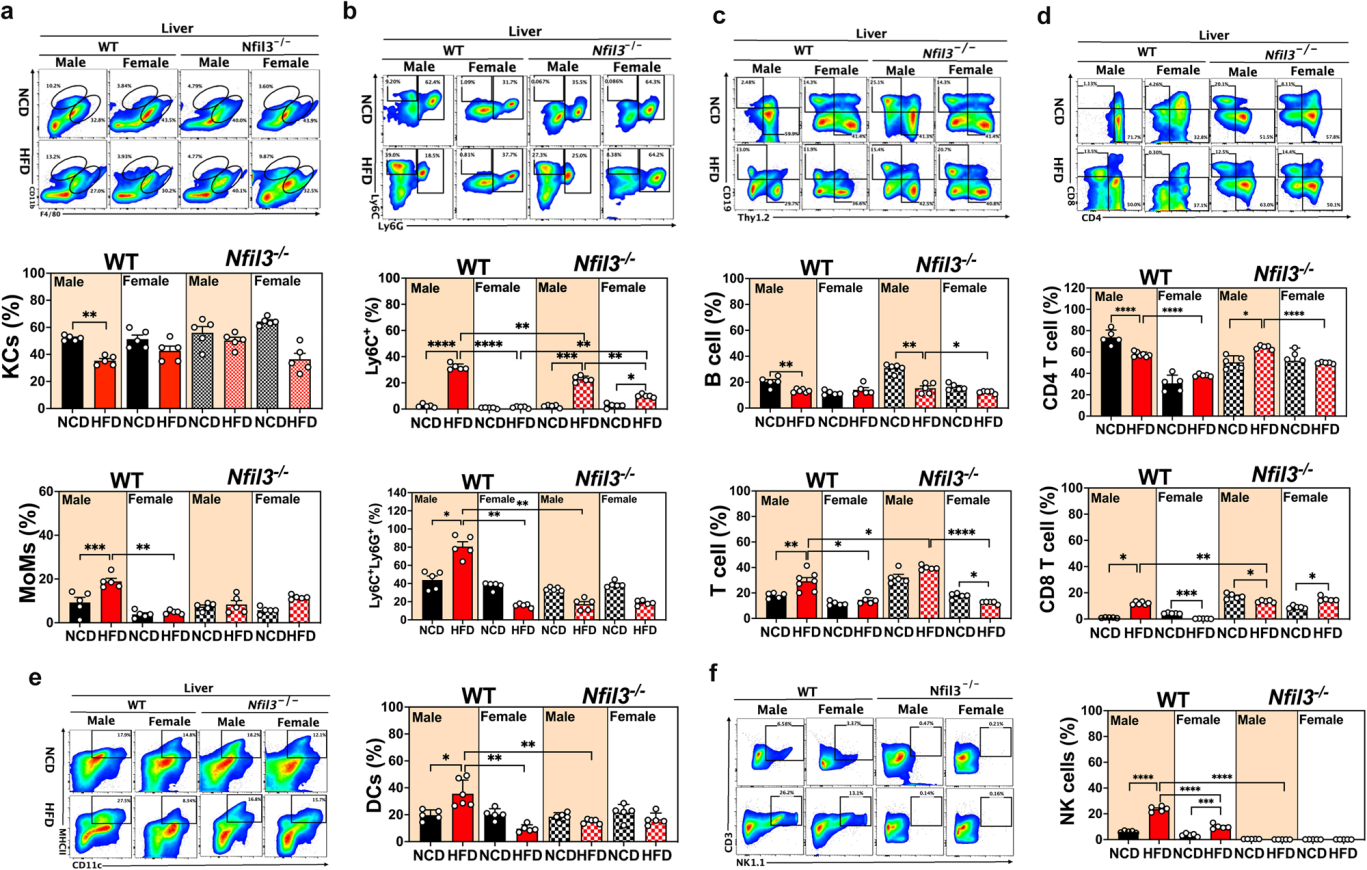
Innate and adaptive immune cell marker expression in the livers of WT and *Nfil3*-deficient (NKO) mice of both sexes fed a HFD. **a** Following animal euthanasia, fluorescent antibodies were used to identify target immune cells, and flow cytometry was used to evaluate the expression of hepatic bone marrow cell markers. Immune cells were separated by CD11b and F4/80 labeling. Statistical analysis of marker expression in CD11b^-^F4/80^+^-labeled Kupffer cells. **b** CD11b^+^, Ly6C^+^, and Ly6G^+^ immune cells were isolated. Monocyte-derived macrophages that are CD11b^+^Ly6C^+^Ly6G^+^. **c** Following animal euthanasia, fluorescent antibodies against CD19 and Thy1.2 were used to identify target cells of immunity, and flow cytometry was used to evaluate the expression of hepatic lymphocyte markers. Statistical analysis of B/T cell ratios in the liver. **d** Target T cells were identified using fluorescent antibodies against CD4 and CD8, and hepatic lymphocyte marker expression was analyzed using flow cytometry. Statistics regarding CD4^+^ helper and cytotoxic CD8^+^ T cells in the liver. **e** Dendritic cells (DCs) were identified using fluorescent antibodies against MHCII and CD11c, and liver DC marker expression was measured using flow cytometry. Expression statistics for hepatic DCs. **f** NK cells were identified using fluorescent antibodies against CD3 and NK1.1, and liver NK marker expression was measured using flow cytometry. Expression statistics for hepatic NKs. The experimental data are presented in standard error of the mean (SEM) format. The following statistical differences are indicated: *P < 0.05; **P < 0.01; ***P < 0.001; ****P < 0.0001.

Male WT-HFD mice had fewer Kupffer cells (KCs) (characterized as F4/80^high^CD11^low^) in the liver than female WT-HFD mice (Fig. 3a). Female WT-HFD mice had higher numbers of KCs-Clec4f^+^ and KC-MHCII^+^ cells than male WT-HFD (Supplementary Fig. S3a). Male mice had a higher proportion of inflammatory Ly6C^+^ monocytes than female WT-HFD mice (Fig. 3b). Male WT-HFD mice had more liver and splenic dendritic cells (DCs) than female WT-HFD mice (Fig. 3e and Supplementary Fig. S3b). Next, we examined the adaptive immunological responses of hepatic B and T cells. CD19 and Thy1.2 was used to identify B and T cells in the liver and spleen, respectively. Male WT-HFD mice had fewer hepatic B cells and more hepatic T cells than female WT-HFD mice (Fig. 3d and Supplementary Fig. S3c). CD4 and CD8 cells in the liver and spleen were used to distinguish between helper and cytotoxic T cells. Male WT-HFD mice had more CD8^+^ T cells and NK cells than female WT-HFD mice. Male NKO-HFD mice had more KCs and CD4^+^ T cells, and fewer inflammatory Ly6C^+^ monocytes, DCs, NK cells, and T cells than WT mice (Fig. 3e, f and Supplementary Fig. S3d). Together, these results revealed that *Nfil3* deletion decreased expression of immune cell markers, enhanced the immunological milieu of the liver, and decreased inflammation in HFD-induced MASLD mice. These findings indicate that *Nfil3* plays a detrimental role in HFD-induced liver fibrosis.

### *Nfil3* deletion protects mice of both sexes against an HFD-induced GM composition alteration

**Fig. 4:**
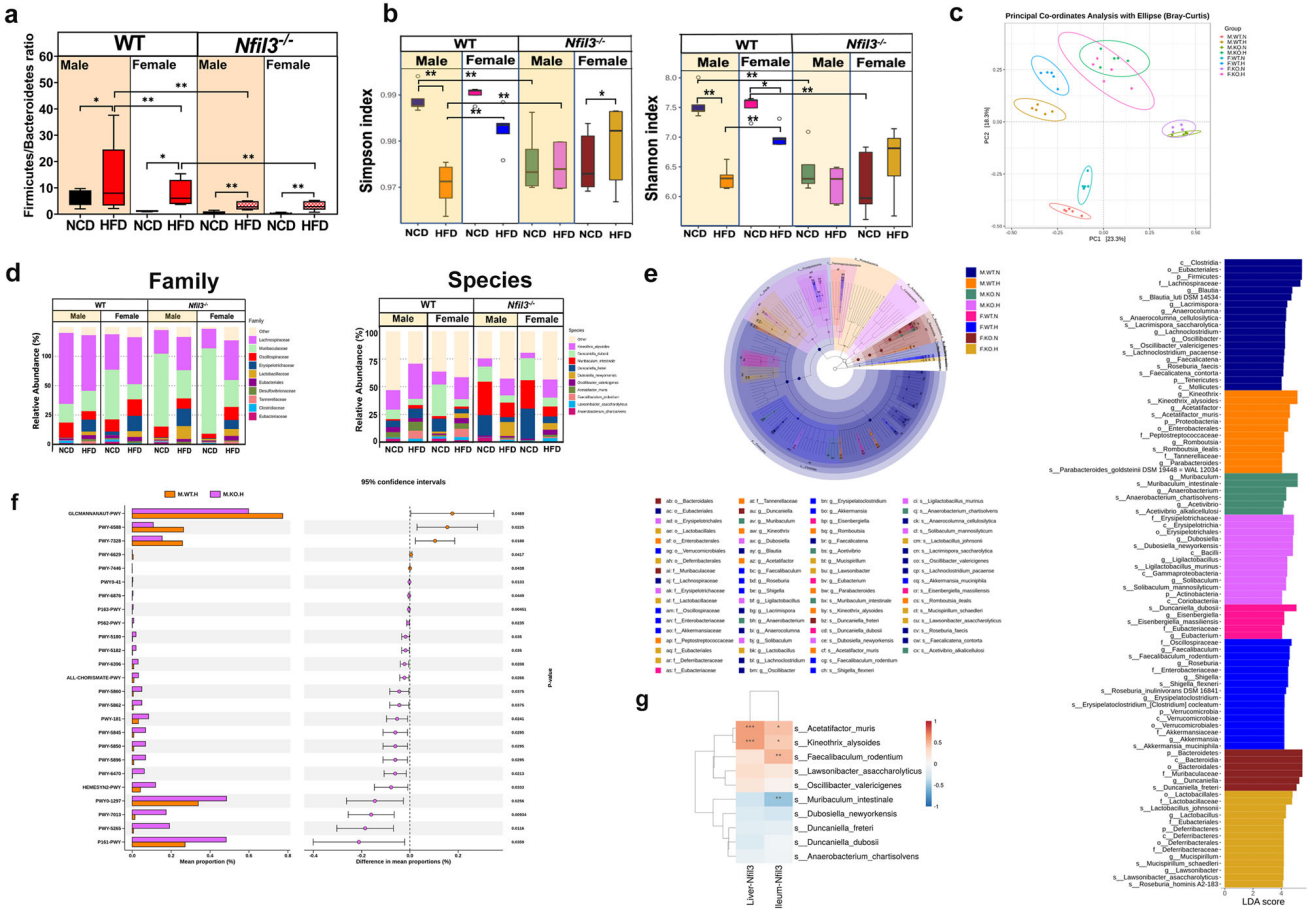
High-fat diet (HFD)-fed WT and *Nfil3*-deficient (NKO) mice exhibit gut microbiota (GM) alterations. **a** Firmicutes to Bacteroidetes ratio in HFD-fed male and female WT and NKO mice. **b** Alpha diversity, Simpson indices, and Shannon indices. **c** The Bray–Curtis analysis leverages common microbial abundance across categories to give values between 0–1. Near-zero microbes are similar. PC1 and PC2 vary in percentages, but distances are maintained. The graph illustrates the 10 species with the greatest relative abundance differences among groups (x-axis, categories; y-axis, relative abundance). **d** Top 10 families with significantly varied relative abundances in each group: the horizontal axis shows groups, vertical axis displays relative abundance (%). Top 10 species with significantly differing relative abundances (x-axis, groups; y-axis, relative abundance [%]). **e** The cladogram illustrates phylum-to-species hierarchical classification as concentric rings from the center. Each small circle’s diameter on different levels shows its category and abundance. The group colors biomarkers that vary significantly from yellow. Red group nodes represent major microbial communities. The graph lists biomarker species. LDA bar plot species with LDA scores >4. Category biomarkers vary statistically. LDA sets bar length. **f** The left figure shows the average abundance ratio of two fundamentally different pathways. The right graph displays group confidence interval differences. Each circle has a lower limit of the 95% confidence interval for the difference in means on the left and the upper limit on the right. Circle Center is a mean difference. Circle color is the highest mean at 0.05 (https://metacyc.org/). **g** Gut microbiota (GM) species and liver and ileum *Nfil3* gene expression in HFD-fed male and female WT and NKO mice. The heatmap depicts *Nfil3* gene expression and the top 20 GM. Acetatifactor_muris, Kineothrix_alysoides, and faecalibaculum_rodentium upregulate liver and ileum *Nfil3*. Red lines represent positive microbial species-value correlations, whereas blue lines suggest negative correlations. Line thickness denotes correlation strength. Groups tested five mice. Asterisks denote statistical differences: Each group has five mice. **0.001 < P < 0.01; ***P < 0.001.

Male WT-HFD mice had a higher ratio of *Firmicutes* to *Bacteroidetes* (F/B) than female WT-HFD mice. Male NKO-HFD mice exhibited a significantly lower F/B ratio than male WT-HFD mice. NKO mice exhibited reduced F/B ratios compared with WT mice (Fig. 4a). Following alpha diversity analysis, Shannon and Simpson diversity indices indicated that male WT-HFD mice had reduced GM diversity compared with female WT-HFD mice. However, male NKO-HFD mice did not show increased alpha diversity compared with male WT-HFD (Fig. 4b). Therefore, *Nfil3* may influence GM diversity. Beta diversity studies revealed group-specific differences in the GM composition. Principal coordinate analysis revealed that the GM of WT mice differed between the HFD and NCD groups. In NKO mice, dietary variations altered the GM composition, and male and female mice had comparable microbiota (Fig. 4c). This indicates that the influence of diet on GM composition may be greater than that of sex, resulting in the similarities between male and female mice. We analyzed the top 10 substantially distinct family- and species-level microbial compositions between the groups (Fig. 4d). The statistical outputs of LEfSe included linear discriminant analysis (LDA) bar graphs, cladograms (distribution of phylogenetic trees), and abundance comparison diagrams of biomarkers that exhibited significant differences between the groups. All groups were subjected to simultaneous and pairwise LEfSe analysis (Fig. 4e). Next, PICRUSt predicts the functional composition of a microbial community based on its taxonomic structure, using a gene family reference database and a phylogenetic tree. Male WT-HFD mice exhibited a higher metabolic pathway (1CMET2-PWY) than male NKO-HFD mice. Male NKO-HFD mice showed higher levels of l-valine biosynthesis (VALSYN-PWY) than male WT-HFD mice (Fig. 4f and Supplementary Fig. S6a). Two species, *Kineothrix alysoides* and *Acetatifactor muris*, were significantly abundant in male WT-HFD mice. *K. alysoides* and *A. muris* counts decreased substantially in male NKO-HFD mice, whereas *Muribaculum intestinale* and *Dubosiella newyorkensis* counts increased significantly compared with those in male WT-HFD mice. These results demonstrate that the absence of *Nfil3* alters the gut microbial composition. Based on these findings, we established the relationship between *Nfil3* expression in the liver and the ileum and the GM. According to heatmap analysis, when expressed in the liver or ileum, *Nfil3* exhibited a statistically significant positive correlation with *K. alysoides*, *A. muris*, and *Faecalibaculum rodentium*. *M. intestinale* and *D. newyorkensis* exhibited a negative correlation with *Nfil3* expression in the liver and intestine (Fig. 4g).

### *Nfil3* deletion protects mice of both sexes against HFD-induced altered GM that regulate phenotypes associated with MASLD

To further elucidate the role of *Nfil3*, BAs, and the GMS in progressively severe MASLD, a multi-omic study of feces was conducted. This included 16 S rRNA sequencing, microbial transcriptomics, and metabolomics in a cohort with varying MASLD phenotypes. First, the relationships between GM, BW, ALT, AST, TG, and Cholesterol were investigated. The positive correlations between *K. alysoides, A. muris*, and *F. rodentium* suggest that these microorganisms promote nutrient absorption and weight gain. The association between these bacteria and elevated alanine transaminase (ALT) and aspartate transferase (AST) levels suggests that the gut-liver axis may be responsible for liver inflammation (Supplementary Fig. S5a). Furthermore, *K. alysoides, A. muris*, and *F. rodentium* elevated low-density lipoprotein (LDL) and cholesterol (CHO) plasma concentrations. These findings indicate that a long-term HFD induces dysbiosis which promotes microbe proliferation that is in turn associated with obesity, resulting in liver dysfunction and elevated blood lipid levels. Increased *M. intestinale* and *D. newyorkensis* abundance in NKO mice (particularly in males) did not cause liver dysfunction or blood lipid alterations. (Supplementary Fig. S5a) *D. newyorkensis* abundance was associated with increased CHO levels in NKO mice. *K. alysoides, A. muris*, and *F. rodentium* were positively associated with *SRB1*, *Cd36*, and *Scd1* expression in terms of GM and endogenous fatty acid synthesis, whereas *F. rodentium* was positively associated with PPAR-γ. *D. newyorkensis* was associated with increased blood lipid levels; however, we also found a strong inverse correlation between this bacterium and p-AMPK, which oxidizes lipids and reduces lipid-induced cellular toxicity. *K. alysoides* showed the strongest association with *Il-6* and hepatic inflammation (Supplementary Fig. S5b, c). This bacterium induced inflammation and fat cell accumulation. Male WT-HFD mice exhibited elevated levels of p-p38 and p-IKK, which are downstream components of the TLR4-mediated inflammatory response (Fig. 2g). *F. rodentium* and *L. asaccharolyticus* abundances also increased in the GM, which correlated with increased p-p38 and p-IKK levels and exacerbated inflammation. *M. intestinale* negatively correlated with the proinflammatory cytokine Il-6 (Supplementary Fig. S5c), indicating that it is likely not involved in p38- and IKK-mediated inflammation. Neither *K. alysoides* nor *A. muris* was associated with proinflammatory cytokines in the small intestine (Supplementary Fig. S5c). The liver fibrosis gene, *Col1a1*, displayed a robust association with *K. alysoides* and *A. muris*, suggesting that these bacteria may contribute to liver fibrosis (Supplementary Fig. S5d). *D. newyorkensis* abundance in the liver immune microenvironment substantially correlated with CD8^+^ T cells, which may explain why male NKO-HFD mice had a higher liver CD8^+^ expression in T cells than male WT-HFD mice (Supplementary Fig. S5e). Only *L. johnsonii* and *L. murinus* were positively correlated with Il-6 expression in the ileum (Supplementary Fig. S5f). Male WT-HFD mice had fewer metabolic pathways than male WT-NCD mice (PWY7242, PWY-6269, et al.). (Supplementary Fig. S6a). Compared with male WT-HFD mice, male NKO-HFD mice had elevated *M. intestinale* and *D. newyorkensis* abundances (Supplementary Fig. S6b). Female WT-HFD mice GM showed a higher prevalence of *Roseburia inulinivorans DSM 16841* and *D. newyorkensis* than the GM of male WT-HFD mice (Supplementary Fig. S6c). Male NKO-HFD mice GM contained more abundant *Solibaculum mannosilyticum* than the GM of female NKO-HFD mice (Supplementary Fig. S6d).

### *Nfil3* deletion protects mice of both sexes against HFD-induced BA profile alterations

**Fig. 5:**
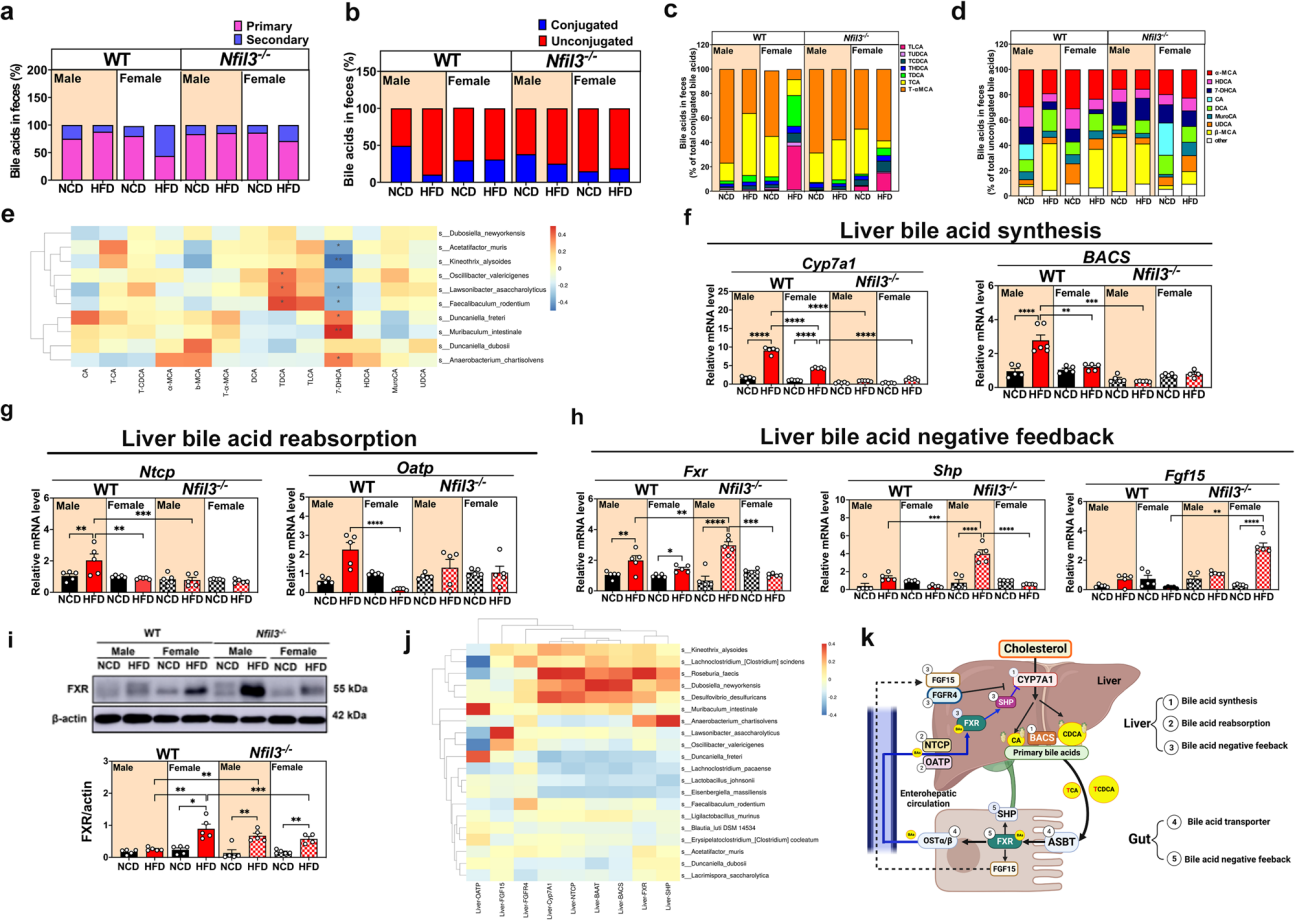
HFD-fed WT and *Nfil3*-deficient (NKO) mice have sex-specific bile acid (BA) levels. **a** Primary and secondary BA percentages in feces of WT and NKO mice of different sexes. **b** Conjugated and unconjugated BA% changes between WT and NKO mice by sex. **c** Conjugated BAs in WT and NKO mouse cecal feces. Male and female WT and NKO mouse taurocholic acid (TCA), tauro-muricholic acid (T-MCA), taurodeoxycholic acid (TDCA), and taurolithocholic acid (TLCA) levels. **d** Unconjugated BA percentages in WT and NKO male and female mouse feces. **e** Cecum BA composition and GM species in male and female WT-HFD and NKO mice. The BA regulatory gene expression heatmap shows the top 20 GM species. Redder colors imply positive GM species-gene correlations, whereas bluer hues suggest negative correlations. TLCA and TDCA enhanced the abundance of particular bacteria in WT mice fed an HFD but decreased that of bacteria in NKO mice. *M. intestinale* abundance was positively associated with 7-DHCA. **f** Liver BA synthesis WT-HFD and NKO mice. In WT-HFD and NKO mice of various sexes, Cyp7a1 limits hepatic BA production. Male WT-HFD mice showed upregulated *Bacs* compared with male WT-NCD mice. **g** Reabsorption of liver BA by *Ntcp* and *Oatp* gene expression in male WT and NKO mice of both sexes. **h** Liver BA negative feedback involving *FXR, Shp*, and *fgf15*. WT-HFD and NKO mice of various sexes express *Fgfr4*. WT and NKO mice of different sexes generate BAs under HFD, while GM do not. **i** Analysis of the FXR protein expression in the liver. **j** The heatmap shows the top twenty genetically modified organism (GMO) species and the gene-regulating BA. Positive microbial-gene interactions are redder, whereas negative ones are bluer. *Cyp7a1, Baat*, and *Bacs* were strongly linked with *Dubosiella newyorkensis* in NKO mice, whereas *Shp* showed modest correlation. *Oatp* correlated positively with *Muribaculum intestinale* and *Duncaniella fretii*. **k** An illustration of the summary. The schematic diagram was created with BioRender.com. *0.01 < *P* < 0.05; **0.001 < *P* < 0.01; ***P < 0.001. Experimental results are presented as mean ± standard error of the mean (SEM). *P < 0.05; **P < 0.01; ***P < 0.001.

Primary BAs increased in the HFD group, particularly in male WT-HFD mice, whereas secondary BAs were decreased relative to male WT-NCD mice. Male NKO-HFD mice exhibited normal secondary BA levels, while female WT-HFD mice had higher secondary BA concentrations than male WT-HFD mice, and female NKO-HFD mice had lower secondary BA concentrations than male NKO-HFD mice (Fig. 5a). Additionally, we evaluated unconjugated BAs and found that male WT-HFD mice had higher unconjugated BA levels than female WT-HFD mice (Supplementary Fig. S7c and Fig. 5b).

Next, we examined BAs in the ileum. Tauroursodeoxycholic acid (TUDCA), taurolithocholic acid (TLCA), and taurodeoxycholic acid (TDCA) levels increased in the ilea of female WT-HFD mice, whereas these levels decreased in the ilea of female NKO-HFD mice. Male WT-HFD mice exhibited higher taurocholic acid (TCA) and Tauro-α-muricholic acid (TαMCA) concentrations than male NKO-HFD mice (Fig. 5c, d), indicating an association between *Nfil3* and primary BA synthesis. 7 alpha-dihydroxy-5 beta-cholestanoic acid (7-DHCA) levels without conjugation were elevated in male NKO-HFD mice (Fig. 5e). The secondary BAs, TLCA and TDCA, were positively correlated with bacterial increase in male WT-HFD mice but negatively correlated with bacterial increase in male NKO-HFD mice (particularly bacteria that were less abundant in the male WT-HFD group) (Fig. 5e). This result suggests that bacteria, such as *Lawsonibacter asaccharolyticus* and *F. rodentium*, convert primary BAs into toxic secondary BAs. *M. intestinale* bound to 7-DHCA (Fig. 5e). Cholic acid (CA) was dehydrated seven times to yield 7-DHCA. This result demonstrated that male NKO-HFD mice contained more 7-DHCA than male WT-HFD mice in the unconjugated BA components.

Liver *CYP* mRNA expression and the ileal *FXR* signaling pathway were investigated. The levels of liver mRNA coding for Cyp7a1, the limiting enzyme in BA synthesis, and *Bacs* (Fig. 5f) and *Baat* (Supplementary Fig. S7a), which conjugates BAs with glycine or taurine, were significantly higher in male WT-HFD mice than in male NKO-HFD and female WT-HFD mice. This result demonstrated that individuals with the WT-HFD phenotype had higher levels of BA gene expression. Male WT-HFD mice expressed significantly more *Cyp7a1*, *Bacs*, and *Baat* than male NKO-HFD mice. This result indicated that the absence of *Nfil3* may alter BA synthesis.

### *Nfil3* deletion protects mice of both sexes against HFD-induced BA reabsorption and liver feedback regulation

**Fig. 6:**
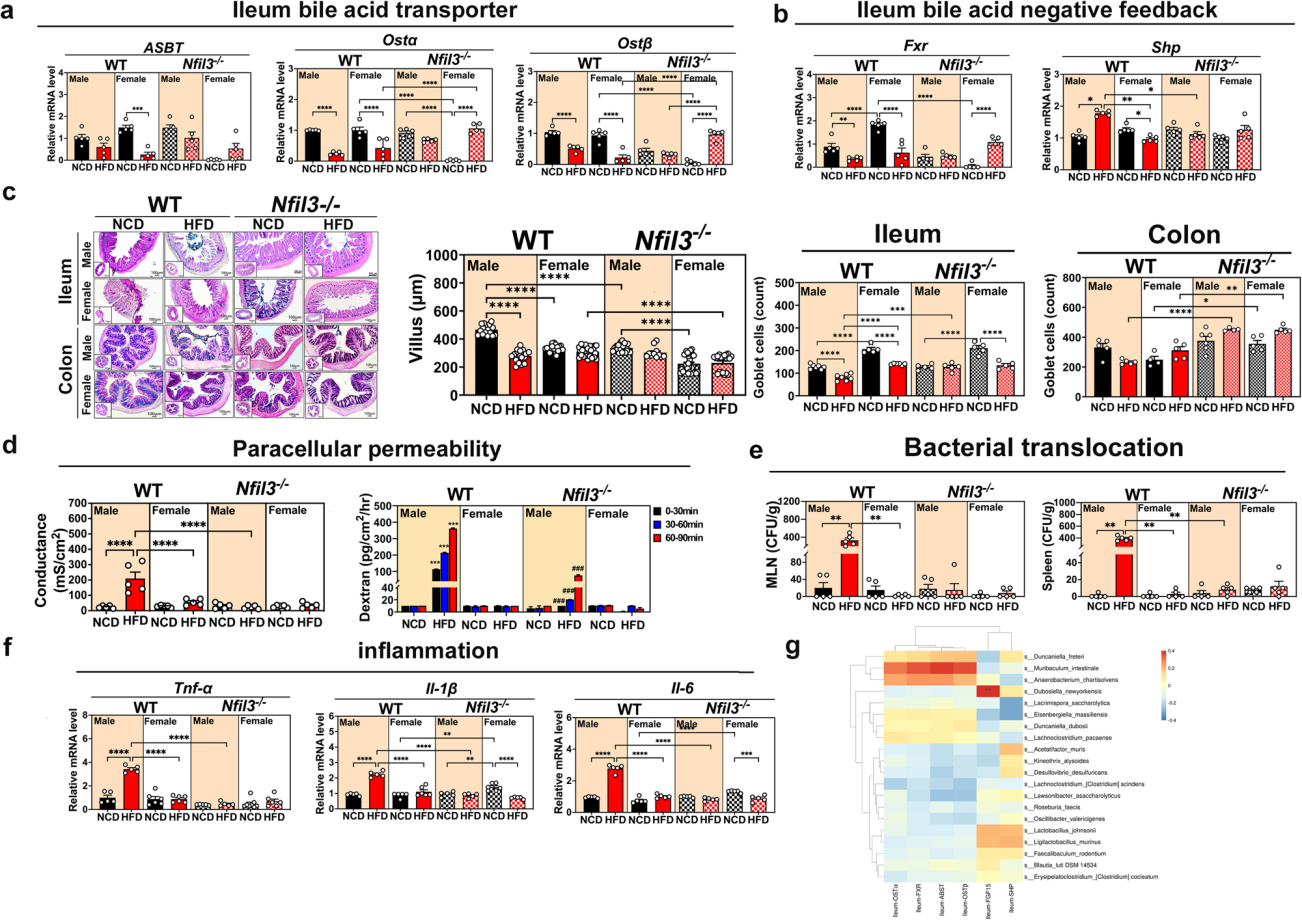
The ileum bile acid (BA) transporter, negative feedback, and paracellular permeability of male and female WT and *Nfil3*-deficient (NKO) mice fed a HFD. **a** After receiving an HFD, WT and NKO mice had different ileum *Asbt, Ost*, and *Ost* expression. *Asbt*, ileum apical membrane BA transporter. Ileum basolateral membrane BA transport proteins *Ost* and *Ost*. **b** After HFD ingestion, WT and NKO mice’ ileum *FXR* and *Shp* genes regulated BA feedback differently. **c** Alcian blue/Periodic acid–Schiff (Ab/PAS) staining shows intestinal changes in HFD-fed WT and NKO mice (scale = 100 μm). The bottom left picture is enlarged 40×, while the larger image is amplified 100×. The top two rows show the small intestine, while the bottom two show the colon. Ileum villus length statistically. Ileum goblet cell count statistics. Statistical colon goblet cell analysis. **d** FITC Dextran 4000 assessed structural integrity-based GB permeability. Dextran 4000 permeability over 90 min. **e** Bacterial translocation (BT) in mesenteric lymph nodes of HFD-fed WT and NKO male and female mice. Mesentery and spleen lymph node colony forming unit (CFU)/mL assesses BT. **f** Tnf-*α*, Il-1*β*, and Il-6 gut inflammation in HFD-fed WT and NKO. **g** Gut microbiota (GM) species and ileum BA transporter and feedback-related gene expression in HFD-fed male and female WT and NKO mice. The BA regulatory gene expression heatmap shows the top 20 GM species. Redder colors indicate more positive correlations between microbial species and their genes, whereas bluer hues indicate stronger negative correlations. *Muribaculum intestinale* in NKO mice positively correlates with ileum *Ost/, Asbt*, and *FXR* expression. *Dubosiella newyorkensis* and *Fgf15* are highly correlated. The standard error of the mean (SEM) is used to present experimental data. * Indicates a statistically significant difference from the NCD group: *P < 0.05; **P < 0.01; ***P < 0.001. # Denotes a statistically significant difference in comparison to the male WT-HFD group: # *P* < 0.05; ## *P* < 0.01; ### *P* < 0.001.

The FXR receptors in the ileum and liver inhibit BA production by reabsorbing BAs from the ileum. Male WT-HFD mice expressed higher levels of the liver BA reabsorption genes, *Ntcp* and *Oatp*, than male WT-NCD and female WT-HFD mice. *Ntcp* and *Baat* mRNA expression levels did not differ between the NCD and HFD groups in male and female NKO mice, whereas *Oatp* expression was decreased (Fig. 5g). Male and female WT-HFD mice showed higher hepatic *FXR* expression than male and female WT-NCD mice, and male NKO-HFD mice exhibited higher hepatic *FXR* expression than male NKO-NCD mice. Male WT-HFD mice displayed marginally lower *Shp* expression than male NKO-HFD mice. Female NKO-HFD mice exhibited higher *Fgf15* expression than female NKO-NCD mice (Fig. 5h). Male NKO-HFD mice exhibited higher *Fgfr4* mRNA levels than male WT-HFD mice (Supplementary Fig. S7b). Female WT-HFD mice had higher FXR protein levels than female WT-NCD and male WT-HFD mice, whereas male and female NKO-HFD mice had higher hepatic FXR protein levels than male and female NKO-NCD mice (Fig. 5i). These data suggest that the lack of *Nfil3* results in an increase in the BA synthesis feedback inhibition upon feeding on an HFD (Fig. 5k). Accordingly, there was also a significant decrease in ileal *Shp* mRNA levels, suggesting fibroblast growth factor-15 pathway downregulation in NKO-HFD mice (Fig. 6b). Moreover, we found a significant increase in *FXR* mRNA levels in the livers of NKO mice (Fig. 5h). This was associated with higher hepatic expression of the *FXR* target gene *Shp* (Fig. 5h), which was involved in lipid metabolism and correlated with decreased steatosis in NKO-HFD mice (Fig. 2a, c). This suggests that females may generate fewer BAs because of greater BA feedback control. These findings indicate that *Nfil3* impairs BA feedback control. An HFD boosts BA production in male WT-HFD mice, but expression of *Fgfr4* cannot be suppressed by the deletion of *Nfil3*.

### *Nfil3* deletion protects mice of both sexes against HFD-induced BA reabsorption in the Ileum

Next, we evaluated ileum BAs transport gene expression. First, compared to male WT-NCD mice, the expression of *Asbt*, which encodes the apical membrane transporter in the small intestine, was lower in both male and female WT-HFD (Fig. 6a). Similar to *Asbt*, both males and females fed the WT-HFD exhibited lower levels of *Ost* and *Ost* expression, both of which encode basolateral membrane transporters (Fig. 6a). Females WT-NCD had increased ileal *FXR* gene expression compared to that in male WT-NCD and female WT-HFD mice. In addition, female NKO-HFD mice showed increased ileal *FXR* gene expression compared to that in female NKO-NCD males. In contrast, male WT-HFD mice expressed more *Shp* than male WT-NCD (Fig. 6b). Based on these findings, females lacking the *Nfil3* gene may produce less *Shp*, preventing *Asbt* from reducing intestinal cell BAs uptake and promoting *Ost* BAs reabsorption. Consequently, enterohepatic circulation recycles BAs back into the liver, thereby enhancing the negative feedback effect.

### *Nfil3* deletion protects mice of both sexes against HFD-induced GB dysfunction and inflammation

Figure 6c and Supplementary Fig. S4a, b illustrate how sex and *Nfil3* deletion alter intestinal tissues. HFD-induced inflammation promotes gut permeability. The gut permeability of WT males was greater than that of WT females. The GB of the NKO mice was less permeable to fluorescent dextran (Fig. 6d). Bacterial translocation (BT) confirmed the GB breakdown. WT males on the HFD had greater BT, as measured using spleen or mesenteric lymph node culture plate colony counts. The culture plates of WT females produced fewer colonies. After HFD feeding, bacteria from NKO mice colonized the culture plates more slowly than those from WT males (Fig. 6e). In WT mice, the GB disintegration due to the HFD caused BT in peripheral organs, whereas *Nfil3* gene knockout reduced this risk. The expression of gut inflammatory factors was used to assess inflammation. Male WT-HFD mice had higher *Tnf-*α*, Il-1β*, and *Il-6* mRNA levels than male WT-NCD mice. *Nfil3* modulated specific inflammatory signals, thereby decreasing intestinal inflammation in NKO mice (Fig. 6f). WT-HFD males had more ileal and colonic DCs compared to NKO-HFD males (Supplementary Fig. S4c, d). Therefore, *Nfil3* deletion prevented changes in the intestinal structure of HFD-fed mice.

### Correlation between the BA pool profile, BA synthesis, and GM composition

*D. newyorkensis* was significantly correlated with *Cyp7a1*, *Baat*, and *Bacs*, which are involved in BA synthesis and feedback regulation, compared with *K. alysoides*. Analysis of the inhibitory factor, *Shp*, revealed that *D. newyorkensis* regulated BAs synthesis in NKO mice. *M. intestinale* and *Duncaniella freteri*, which were highly expressed in the NKO group fed with an NCD, were positively correlated with *Oatp* in the liver. These bacteria regulate BA reabsorption by the liver. *Ost, Asbt*, and *FXR* were positively correlated with *M. intestinale*, whereas *Fgf15* was highly positively correlated with *D. newyorkensis* (Fig. 5j), confirming that these bacteria enhanced BA reabsorption in NKO mice while decreasing BA synthesis. Additionally, a positive correlation was identified between *Muribaculum intestinale* and ileum expressed *Fxr*, *Ostα/β*, and *Asbt*. *Dubosiella newyorkensis* exhibited a highly positive correlation with *Fgf15* (Fig. 6g). This finding provides additional evidence for the enhanced bile acid reabsorption capacity of *Dubosiella newyorkensis* and *Muribaculum intestinale* in Nfil3-/- mice, as well as their ability to inhibit excessive bile acid synthesis.

## Discussion

We demonstrated the critical role of *Nfil3* in MASLD progression and SDs in a GM-dependent manner. Furthermore, we found that *Nfil3* deficiency modulated GM diversity and directly increased *M. intestinale*-*D. newyorkensis*-mediated 7-DHCA levels to suppress secondary BA biosynthesis, which in turn activated hepatic *Nfil3-FXR-FGF15* signaling to ameliorate MASLD.

The GM metabolizes multitudes of molecules, including BAs and short-chain fatty acids (SCFAs), many of which affect host physiology and diseases. In this study, we established that BAs may play a role in *Nfil3* regulation of MASLD with SDs. Notably, *Nfil3* regulated BA metabolism and increased 7-DHCA levels in secondary BA biosynthesis. Increasing primary BA levels result in a dramatic shift toward *Firmicutes* abundance, particularly of *Clostridium cluster XIVa*, as well as an increase in deleterious secondary BA deoxycholic acid (DCA) production (18). In this study, male NKO mice exhibited increased primary BA and decreased secondary BA levels. In addition, the *M. intestinale* and *D. newyorkensis* abundances were positively associated with 7-DHCA secondary BA concentrations. These findings imply that DCA and BA profiles can be used as prognostic and disease-monitoring biomarkers. Furthermore, these findings support further studies for these indications.

*Nfil3* regulates BA production under HFD feeding and induces the liver to produce BAs from dietary fat, resulting in an increase in the proportion of primary BAs^18^. WT mice fed an HFD had higher concentrations of major BAs, particularly TCA and T-MCA^18^. Reduced primary BA levels were observed in the NKO mice. As anticipated, secondary BA levels increased the most in female WT mice fed an HFD. Generally, the GM of female mice is more diverse than that of male mice, and secondary BA levels increase with age^19^. In our study, WT female mice had higher levels of secondary BAs, TLCA, and TDCA^20^ and a greater intestinal bacterial species diversity. Secondary BA reabsorption into the liver results in TDCA and TLCA production. Correlation of TDCA and TLCA with *F. rodentium* abundance indicated that *F. rodentium* expressed BAs hydrolase (BSH), which removes glycine or taurine from conjugated primary BAs and transforms them into secondary BAs that are reabsorbed into the conjugated liver. These bacteria may also stimulate secondary BA production in the gut, followed by BA reabsorption in the liver. In other studies, mice administered *Gastrodia elata* blume extract after an HFD had a greater abundance of *F. rodentium* and a robust association with TDCA^21^. The FXR and TGR5 agonist, TDCA^22^, stabilizes BA production and increases GLP-1 secretion to maintain glucose homeostasis^23^. This may explain why WT female mice with higher TDCA levels were better able to maintain stable blood glucose levels and reduce BA synthesis-induced adiposity than male mice. These findings provide evidence that the presence of *Nfil3* during long-term HFD increases harmful GM composition, resulting in intestinal and liver-related diseases. However, in the absence of *Nfil3*, the GM was altered with an increase in anti-obesity and anti-inflammatory bacterial abundance. Probiotic studies have examined both the *Lactobacillus* strains*: L. johnsonii* and *L. murinus*. *L. johnsonii* can survive in stomach acids and bile salts in animal hosts. This bacterium ferments lactic acid and short chain fatty acids, which may thicken the host gut mucosal barrier, inhibit pathogenic bacteria, control inflammation, and lower blood lipid levels^24,25^. MASLD-related and decreases HFD-induced liver damage and steatosis are improved by probiotic^26^. In the murine intestine, *L. murinus* suppresses infections and generates antibiotics^27,28^.

BAs interact with cellular BA receptors. FXR activation positively regulates glucose homeostasis, lipid metabolism, hepatic inflammation, and liver fibrosis during MASH progression^29^. FXR, a member of the nuclear receptor superfamily, plays an important role in the metabolism of glucose, TGs, and BAs^30,31^. Activated FXR in the ileum induces fibroblast growth factor 15/19 production (FGF15/19), which inhibits rate-limiting enzyme expression for hepatic BA synthesis and CYP7A1 in the liver^32^. Our study found that in male WT-HFD, FXR protein expression decreased in the intestinal and liver tissues and inhibited *Shp* expression, which ultimately resulted in *Cyp7a1* and *Bacs* upregulation in hepatocytes. TG levels increased after PPAR-γ, and *Cd36* positively regulated the expression of genes (*Fasn, Acc1*, and *Scd1*) responsible for lipogenesis. Thus, the role of FXR signaling in non-alcoholic fatty liver disease pathogenesis has been determined.

Hepatic *Nfil3* expression inhibits AMPK activation and promotes fat accumulation in HFD mice. AMPK regulates energy and inhibits *Acc1*, which synthesizes fatty acids, activates mitochondrial oxidation, and uses fat to generate ATP^33,34^. In our study, p-AMPK expression decreased in male WT-HFD mice, whereas female mice indicated no change in p-AMPK expression. Blood CHO levels in NKO mice were comparable to those in WT mice, indicating that dyslipidemia was not reduced. Further studies on GM suggested a correlation between elevated levels of *D*. *newyorkensis* in NKO mice and the blood lipoprotein CHO. A strong correlation was observed between *D*. *newyorkensis* and AMPK activity. *D*. *newyorkensis* reduces oxidative stress and increases inflammation. This bacterium has the potential to treat obesity, hypertension, and liver disorders when administered as a probiotic (43, 44). In numerous experiments, MASLD symptoms were alleviated in the NKO mice. *Tnf-α*, *Il-1β*, and *Il-6* promote hepatic inflammation in MASLD^35^. *Tnf-*α, *Il-1β*, and *Il-6* gene expression was increased in WT-HFD mice. Sectioning and hematoxylin and eosin staining of liver tissues and elevated alanine aminotransferase and aspartate aminotransferase levels indicated liver injury. In WT-HFD mouse livers, there were more Ly6C^+^ inflammatory monocytes, DCs, and CD8^+^ T lymphocytes compare NKO-HFD mice^36^. In contrast, the numbers of the majority of proinflammatory immune cells decreased in NKO mice, with the exception of CD8^+^ T cells. A positive correlation was established between increased *M. intestinale* bacteria in NKO mice and CD8^+^ T cells.

Our study presents some limitations: First, *Nfil3* is a conventional regulator of innate immunity; therefore, it may be efficacious in immune cells in the pathology of MASLD, which requires further investigation. Second, we demonstrated a substantial *Nfil3* downregulation in obese livers, but the detailed mechanisms underlying the MASLD-related variation in *Nfil3* expression remain unclear. Third, regarding the mechanisms underlying the *Nfil3*-ameliorated FXR signaling pathway, precise MASLD therapy should target the *Nfil3-FXR-FGF15* axis to clarify the molecular events in detail. Fourth, our study has revealed that *Nfil3* deficiency may have major effects on the immune system, but it can also affect other organs and systems in the body. We can research the detrimental effects of *Nfil3* deficiency on additional organs in the future.

**Fig. 7:**
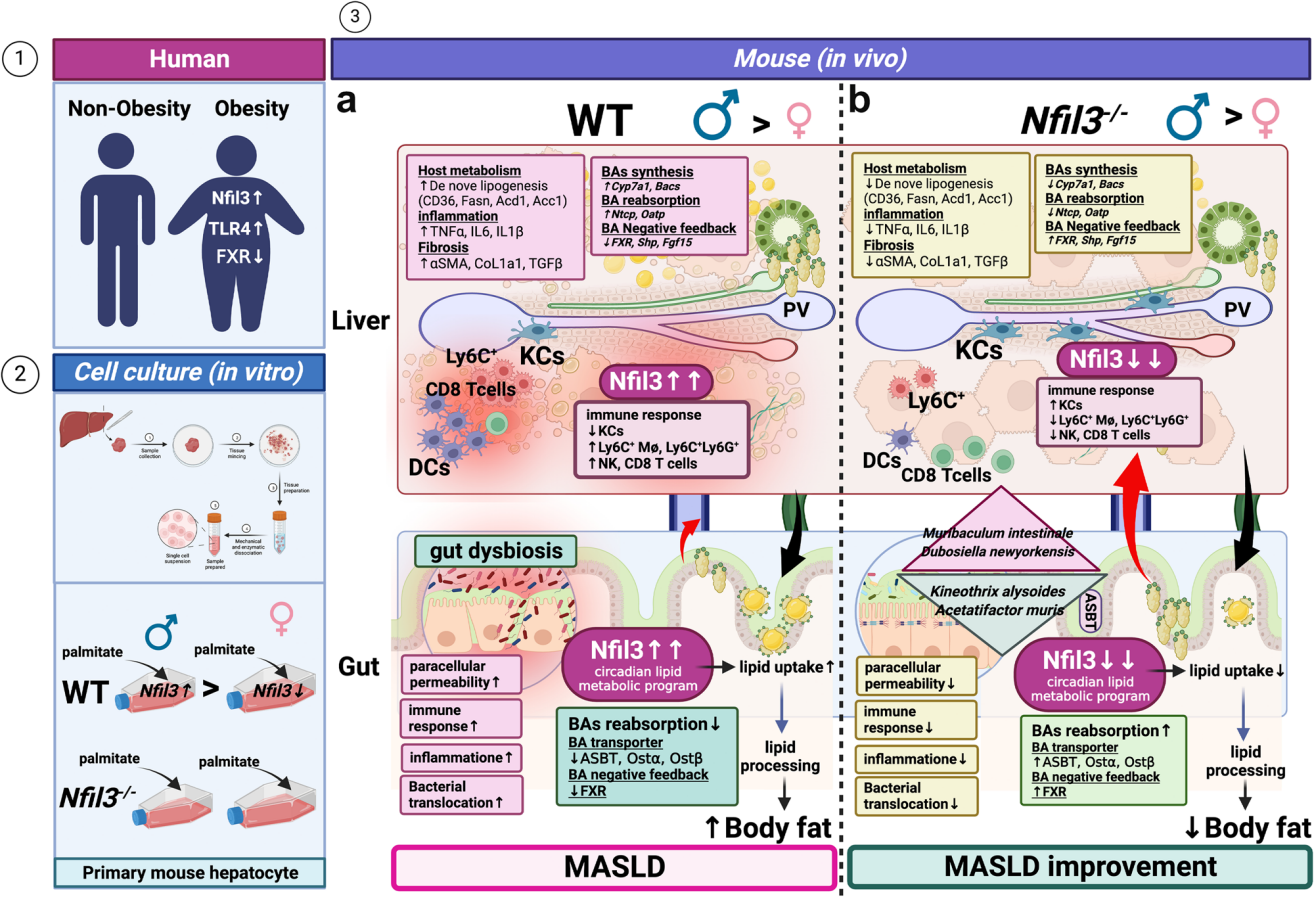
An illustration of the summary. (1). Human Segment: Comparative representation of *Nfil3, TLR4* and *FXR* expression levels in individuals with and without obesity. (2). In Vitro Cell Culture: Depicts the treatment process of primary hepatocytes with palmitate and the subsequent effects on *Nfil3* expression in male and female conditions. Bottom Panels: Show the effects of palmitate on primary mouse hepatocytes in WT and *Nfil3* knockout conditions, including changes in lipid uptake and fat. (3). Mouse In Vivo Liver and Gut Sections: **a** WT Male and Female: Illustrates the impact of *Nfil3* expression on liver metabolism, bile acid synthesis, and immune cell distribution in the liver, as well as gut dysbiosis, and lipid uptake in a WT mouse. **b** *Nfil3* Knockout (*Nfil3*^*-/-*^) Male and Female: Shows the altered metabolic pathways in the liver and gut, immune response, and microbial composition due to the lack of *Nfil3*. Key Outcomes: MASLD (Metabolic Associated Steatohepatitis Like Disease): The figure connects *Nfil3* expression with disease progression or improvement, indicating the importance of *Nfil3* in maintaining metabolic homeostasis and preventing disease. The arrows indicate the directionality of effects or results, with up and down arrows symbolizing an increase or decrease in the associated factors, respectively. The diagram integrates the relationships between host metabolism, bile acid dynamics, immune response, gut microbiota composition, and the resultant metabolic disorders. The schematic diagram was created with BioRender.com.

In conclusion, our results suggest that the lack of *Nfil3* in HFD-fed mice is associated with GM (abundance increase: *M. intestinale* and *D. newyorkensis* and abundance decrease: *K. alysoides* and *A. muris*), resulting in alterations in BA pool composition, affecting BA synthesis regulation via *Nfil3-FXR-Fgf15/fgfr4* signaling, reducing Ly6C^+^ macrophage recruitmƒent, and preventing liver steatosis and inflammation (Fig. 7). *Nfil3* is a novel potential GM-dependent therapeutic target for MASLD treatment. Our results reveal new avenues for determining whether *Nfil3* can enhance the BA-dependent microbiome in progressive MASLD and lead to the development of novel biomarkers and therapeutics.

## Methods

### Patient specimens

Human peripheral blood mononuclear cells (PBMCs) samples were obtained from healthy volunteers and patients with obesity, including men and women were recruited and classified into two groups depending on their body mass index (BMI). A non-obesity group (BMI  <  25 kg/m^2^) was composed of 5 volunteers and an overweight-obese group (BMI  ≥  25 kg/m^2^) of 10 volunteers (5 women and 5 men). The inclusion criteria were subjects aged 18–45 years old, with no chronic disease who did not take regular medication or drugs. To avoid potential bias, both groups included approx. 50% men/women and there was no difference in their average age. The number of individuals has been calculated to provide an 80% power and to detect a difference of 10% in BMI, with a *p*  <  0.05. Informed consent in writing was obtained from all the patients and volunteers before inclusion in this study. All ethical regulations relevant to human research participants were followed. The study IRB conformed to the ethical guidelines and was approved by the Clinical Research Ethics Committee of the National Cheng Kung University Hospital. The Human Ethics Committee accepted this research (IRB No: B-BR-108-018-T) on obesity.

### Animals, diets, and the murine MASLD model

Male and female WT mice were obtained from the National Laboratory Animal Center (Taipei, Taiwan). Hsu et al. obtained NKO mice from the National Laboratory Animal Center and transferred them to the Immunology Institute of the National Taiwan University. Seven weeks prior to the experiment, the animals were housed in a temperature-controlled environment between 20 °C and 24 °C with a 12 h light/dark cycle. Male and female WT or NKO mice were fed a NCD (purchased from the National Yang-Ming University Laboratory Animal Center) or an HFD (Envigo, USA) containing 60.3% fat, 21.4% carbohydrates, and 18.8% protein. After one week of acclimatization, mice were divided into two groups based on diet. The Institutional Animal Care and Use Committee of the National Yang-Ming University approved protocol number 1100902 and 1121209. We have complied with all relevant ethical regulations for animal use. The details of the methods used in this study are described in the Supporting Materials and Methods.

### Fasting blood glucose and oral glucose tolerance test (OGTT)

Fasting glucose levels were measured every four weeks. After a 14 h fast, blood glucose levels were determined from blood collected from a tail incision (0 min data) using test strips and a Taiwanese Super OK meter. The OGTT was performed at 20 weeks. A 25% glucose solution (1 g/kg) was supplied to the mice via a gavage catheter. Blood glucose levels were monitored at 15-, 30-, 60-, and 120 min using incision-site blood glucose strips.

### Biochemistry

Blood samples were tested using the glucose meter Contour®Plus (Ascensia Diabetes Care, Basel, Switzerland) immediately after blood collection. Hepatocellular disintegration and necrosis were assessed spectrophotometrically in serum samples using aspartate aminotransferase (AST) and alanine transaminase (ALT). Cobas®c111Analyzer (Roche Diagnostics GmbH, Penzberg, Germany) recorded 340/378 nm extinction. Plasma tryglyceride (TG) levels were determined using a TG Colorimetric Assay Kit (Nr.:10010303, Cayman Chemical Company, Hamburg, Germany), according to the manufacturer’s instructions.

### Histological analysis

The histopathological organs were stored in 4% paraformaldehyde (PFA) after euthanasia. Tissues were dehydrated in 30%, 50%, 70%, and 90% alcohol for 2 h overnight and in 99% alcohol twice for 2 h. Xylene was used for 30–60 min (product code: 10291531; J.T. Backer, Sweden). After the tissues became translucent, xylene was removed, and paraffin was infiltrated overnight. The tissues were then embedded in paraffin. The paraffin-embedded tissues were cut into 5 µm square sections using a Microtome (OS-315 Rotary; U.K.). The samples were then subjected to an alcohol gradient of 100% for 7 min (twice) and 95% for 7 min. The samples were then left to hydrate for 5 min and washed for 1 min. After staining, the tissue samples were air-dried. Liver tissues were incubated overnight in PFA to obtain frozen sections. After 2 h or overnight incubation, the tissues were treated with an optimum cutting temperature (OCT) chemical (Avantor, Cat 25608-930). Flash-frozen OCT-embedded tissues were frozen at −80 °C.

### Hematoxylin and eosin (H&E) staining

H&E staining was used to examine the tissue nuclei and cytoplasm. Tissue samples were stained with hematoxylin for 1–2 min before washing. Next, they were soaked in eosin dye for 30–60 s before being rinsed under running water (the staining duration varied per tissue). Pure ethanol was then used to lighten the samples, which were consequently observed by microscopy.

### Picrosirius red staining (PRS)

PSR was used to observe fibrosis. Picrosirius red (Micro-Sirius Red, Abcam, ab150681) was applied to the tissue samples for 60 min. They were rinsed twice with acetic acid and again with pure alcohol and microscopically observed.

### Alcian blue-periodic acid-schiff staining (Ab/PAS)

Alcian Blue 8GX (Sigma, A5268-10G) dye was used to clean the tissue samples for 15 min. After 5 min, the color of the Periodic Acid (Abcam, ab150680) was removed. Schiff’s solution was used to rinse the samples for 5–10 min. Hematoxylin-stained cell nuclei were washed after 2–3 min and observed by microscopy (Nexcope NE 910, U.S.A).

### Red O oil staining

Frozen sections were colored, and the OCT was dissolved in frozen tissue slices in PBS and washed with 60% isopropanol (3:1). After 15 min, the slides were stained with Oil Red O working solution (#O0625; stock: ddH2O = 3:2; Sigma) and washed with 60% isopropanol. After hematoxylin staining of the cell nuclei, the transparent samples were rinsed and air-dried.

### RNA extraction

TRIzol (Invitrogen, Carlsbad, CA, USA) (100 µL/0.01 g) was added to intestinal or hepatic tissue. After shearing, a pulverizing device was used to homogenize the tissues and carefully combine them with 100% ethanol. The sample was then centrifuged for 1 min at 12,000 × *g* and 4 °C and the supernatant transferred to a column. RNA was isolated using Direct-zol RNA MiniPrep (ZYMO RESEARCH, R2050). A Nanodrop was used to measure the RNA concentration, and an agarose gel was run at 90 V for 20 min. The RNA was then maintained at −80 °C.

### Complementary DNA (cDNA) transcription

Nuclease-free water was used to modify and normalize RNA concentrations in the samples. A reverse transcription buffer was prepared using high-capacity cDNA reverse transcription reagent kit (ThermoFisher, 4374966). A Bio-Rad T100TM thermal cycler was used to facilitate cDNA transcription. The sample temperature was maintained at −20 °C.

### Quantitative reverse transcription polymerase chain reaction (qRT-PCR)

Total RNA isolation and RT-PCR were conducted using the manufacturer’s instructions. The results were calculated using Ct values and normalized to *GAPDH* mRNA levels. The primer sequences are shown in Supplementary Table S1. After the experimental procedure, statistical analyses were performed based on the calculated Ct values.

### Immunoblot analyses and antibodies

Western blotting was performed using standard methods. Western blotting was conducted following standard protocols. Briefly, protein samples were separated by SDS-PAGE using appropriate percentage gels based on the molecular weight of the target proteins. After electrophoresis, proteins were transferred onto nitrocellulose or PVDF membranes using a semi-dry or wet transfer apparatus. Membranes were then blocked with 5% non-fat milk or BSA in Tris-buffered saline with Tween 20 (TBST) for 1 hat room temperature to minimize non-specific binding. Primary antibodies specific to the target proteins were diluted in blocking solution and membranes were incubated with primary antibodies overnight at 4 °C with gentle agitation. Following primary antibody incubation, membranes were washed several times with TBST to remove excess primary antibody. Next, membranes were incubated with corresponding secondary antibodies conjugated to horseradish peroxidase (HRP) or fluorescent dyes for 1 h at room temperature. After secondary antibody incubation, membranes were washed again with TBST to remove excess secondary antibody. Protein bands were visualized using an enhanced chemiluminescence (ECL) detection system for HRP-conjugated antibodies or a fluorescence imaging system for fluorescently labeled antibodies. Images were captured using appropriate imaging equipment and analyzed using densitometry software to quantify band intensities. The uncropped blots PVDF membrane are depicted in the images showing the uncropped western blots in Supplementary Fig. S8. The following antibodies were used: anti-β-actin (Cell Signaling Technology, 3700), anti-phospho-AMPKα (Cell Signaling Technology, 2535), anti-AMPK (Santa Cruz, sc-25792), anti-PPARγ (Santa Cruz, sc-7273), and anti-FXR (Cell Signaling Technology, 72105). The antibody is shown in Supplementary Table S2. For each experimental condition, appropriate positive and negative controls were included to ensure the specificity and accuracy of the western blotting results.

### Flow cytometry and liver and spleen-infiltrating leukocyte isolation

The liver was perfused with Hank’s balanced salt solution (HBSS [II]) until blood was completely cleared from the portal vein. An infusion of 0.05% collagenase (SI-C5138; Sigma Aldrich) was administered to soften the liver. After removing the liver, the tissue was shredded and submerged in a collagenase solution. A 50 mL centrifuge tube and a 70 µm filter were used to grind the liver and fluids. The tube was then filled with 50 mL PBS. Centrifugation was performed at 50 × *g* for 5 min to separate non-parenchymal cells from hepatocytes. The supernatant containing NPCs was centrifuged at 3000 *g* for 10 min and discarded, and 1 mL of RBC Lysing Buffer Hybri-MaxTM (Sigma Aldrich, #R7757-100ML) was used to lyse the blood cells for 1 min. The lysate was neutralized with PBS in a flow tube. To count the cells, 10 µL of the supernatant was centrifuged at 1800 rpm for 5 min. The samples were then incubated for 10 min with 100 µL of the Fc block and Golgi stop combination. APC bead buffer (65 µL) was added to 100 µL of anti-mouse CD45 APC antibody and incubated for 30 min. iMeg buffer (BD Biosciences, 552362) (1 mL) was added, and the sample was incubated for 8 min. After 4 min of incubation, another 1 mL of iMeg buffer was added to the non-brown region that adhered to the magnetic stand. Using a magnetic support, the liquid was removed from the non-brown zone. Cell subdivision using targeted antibody detection was performed. Before fixing with 2% PFA, 100 µL of the produced antibody was incubated for 30 min. The supernatant was removed, 200 µL PBS was added, and flow cytometry and gating strategy was performed^37^.

### Cell cultures

Isolation of primary mouse hepatocytes (PMHs) was described previously^38^. PMHs cells were treated with DMSO and 400 μM palmitate for 24 h and harvested for RT-qPCR.

### Using chamber intestinal permeability assessment

After longitudinal dilatation of the intestinal section, the waste was carefully removed. The white tank of the instrument contained the intestinal segment with the villous side facing upwards, whereas the second transparent tank enclosed it. Both serosal and mucosal chambers were equipped with two sets of electrodes. The mucosal chamber contained mannitol, whereas the serosal chamber contained glucose. After 800 µL was transported, an equivalent volume of serosal solution was aspirated. The mucosal chamber was then incubated with FITC dextran 4000. The absorbance of 800 µL of serosal solution was measured after 30-, 60-, and 90-min. Electrodes determined the differences in electrical current between tissue extremities. Using the same voltage and Ohm’s law (V = IR) to calculate the resistance (R), the tissue conductivity (G) was calculated as G = 1/R. High G values indicated severe intestinal barrier degradation.

### Permeability assay

The mucosal-to-serosal flux rate of FITC dextran 4000 (44 kDa; Sigma Aldrich, UK) (catalog number: 46944) was used in this assay, and the gut epithelial permeability was determined. After equilibration, 300 µL of 4 kDa FITC dextran was added to the mannitol-Krebs solution. An FITC dextran starting concentration of 1 mg/mL was used. Initial tests revealed that FITC dextran of 4 kDa was not readily detectable in healthy murine tissues. At 0, 30, 60, and 90 min post-luminal addition of FITC dextran, 800 μL samples of serosal buffer were collected and replaced with equal volumes of Krebs buffer/glucose, and fluorescence was measured using TECAN Infinite 200/200 PRO, TECAN Spark. The read emission and fluorescence absorption wavelengths were 488 nm and 520 nm, respectively. The serosal concentrations were determined using a standard curve derived from known concentrations. The paracellular flux was expressed as pmol/cm^2^/h.

### Bacterial translocation

After euthanasia, the spleen and mesenteric lymphoid tissues of the mice were removed and deposited in a microcentrifuge tube with homogenized particles. The net weight of each tissue sample was then determined. For every 0.01 g of tissue weight, 100 µL of sterile PBS solution was added to the tube. The tissues were homogenized using homogenizers. Tissue lysate (200 µL) was distributed on a Scientific Biotech Corporation sheep blood agar plate using a sterile glass rod and the plate was incubated at 37 °C overnight. On the following day, bacterial colonies were evaluated.

### BA analysis

Following euthanasia, the intestinal contents were transported BIOTOOLS Co., Ltd., for liquid chromatography-mass spectrometry BA analysis. Samples (10 mg) were combined with 500 µL of extraction solution containing internal standards (DCA-d6, GCA-d4, and TCDCA-d4). After 4 min of grinding at 2.35 Hz, the samples were vortexed for 30 s. Cold sonication was then performed. After 1 h at −20 °C, the mixture was centrifuged for 15 min at 12,000 rpm and 4 °C. The analytical vessel received a 200 μL supernatant aliquot. The sample was injected into an ACQUITY UPLC BEH C8 (Waters) (1.7 m × 2.1 × 100 mm) column at 60 °C. The target Lynx then combined the signal intensity data with the concentration values.

### GM and diversity analysis

#### Fecal DNA extraction

Five feces samples per group of 2–3 were collected in 2 mL microcentrifuge tubes after a 20-week HFD. Containers were maintained at ×80 °C. Lysate buffer (700 µL) and a pestle were used to homogenize the feces in each collection container. Proteinase K (1 µL) was added and incubated at 65 °C in a dry environment overnight. Chloroform (700 µL) was added to each microcentrifuge container and vigorously mixed until the chloroform became transparent and white. After 15 min of centrifugation at 12,000 × *g*, the aqueous layer was transferred to a new microcentrifuge tube. The sample was then mixed with 700 μL isopropanol, mixed thoroughly, and centrifuged again at 12,000 × *g* for 10 min. The residue was discarded. Ethanol (70%, 200 µL) was added, the particle gently agitated, and centrifuged for an additional 10 min. This step was repeated 200 µL of 100% ethanol. The stool DNA was air-dried to eliminate the ethanol. Finally, 100 µL of TE buffer was added, and the sample was incubated at room temperature or 37 °C dry immersion until the DNA dissolved and stored at −20 °C.

#### Full-length 16 S sequencing analysis

The concentration and purity of the fecal DNA were determined using a NanoDrop spectrophotometer. After validation by BIOTOOLS Co., Ltd., the samples were sequenced. Microbes were analyzed using third-generation 16 S metagenomic sequencing. Universal primers (27 F + 1492 R) were used to amplify the 16 S genes. After quality control, library construction, DNA purification, and damage restoration, third-generation PacBio sequencing systems were used to generate sequences. ASVs are generated and compared to databases such as NCBI, GreenGenes, and SILVA to determine species and abundance. These analyses included diversity, microbiological composition, functional predictions, and correlation. The cloud platform of BIOTOOLS Co., Ltd., organized and analyzed the data. In biological datasets, LEfSe is used to filter genomic characteristics and identify high-dimensional biomarkers. LEfSe can be used to identify biomarkers with statistical significance and compare subgroups. Further, it can simultaneously detect species with substantial variations in abundance across taxonomic levels (phylum, class, order, family, and genus). The algorithm places strong emphasis on statistical significance and biological relevance. PICRUSt was used to predict metagenomic functions using marker genes by estimating the functional potential of microbial communities by inferring the presence and abundance of functional genes from marker genes such as 16 S rRNA sequences. Welch’s *t*-test was used to identify distinct species within each taxonomic hierarchy (phylum, class, order, family, genus, and species). Species exhibiting statistically significant differences (*P* < 0.05) were identified, and a bar graph depicting intergroup species differences was generated.

### RNA extraction from human PBMCs

#### RNA quantification and qualification

An RNA SimpliNanoTM Spectrophotometer (Biochrom, MA, USA) was used to verify RNA purity and quantity. A Qsep 100 DNA/RNA Analyzer (BiOptic Inc., Taiwan) was used to evaluate RNA degradation and integrity.

#### Library preparation for transcriptome sequencing

Transcriptome sequencing library preparation was performed as follows: RNA sample preparations included 1 μg total RNA per sample. Following the manufacturer’s instructions, sequencing libraries were created using the KAPA mRNA HyperPrep Kit (KAPA Biosystems, Roche, Basel, Switzerland), and index codes were applied to assign sequences to the samples. Magnetic oligo-dT beads were used to isolate mRNA from total RNA. High-temperature incubation with magnesium in the KAPA fragment, priming, and elution buffer (1×) treatment fragmented the captured mRNA. Random hexamer priming produced first-strand cDNA. Combined second strand synthesis and A-tailing converted the cDNA: RNA hybrid to double-stranded cDNA, integrated dUTP into the second strand, and added dAMP to the 3′ ends. dsDNA adapters with 3′ dTMP overhangs were ligated to library insert fragments to create adapter-containing fragments. KAPA Pure Beads (KAPA Biosystems, Roche) were used to purify library fragments to select 300–400 bp cDNA fragments. KAPA HiFi HotStart ReadyMix (KAPA Biosystems) and library amplification primers were used to amplify the library with adapter sequences at both ends. The dUTP-marked strands were not amplified, which enabled strand-specific sequencing. Finally, PCR products were purified using KAPA Pure Beads, and the Qsep 100 DNA/RNA Analyzer (BiOptic Inc., Taiwan) was used to evaluate library quality.

#### Data analysis

CASAVA base calling converts high-throughput sequencing data (Illumina NovaSeq 6000 platform) into FASTQ-formatted raw sequenced reads. FastQC and MultiQC^39^ checked the quality of the FASTQ files. Trimmomatic (v0.38)^40^ removed low-quality reads, trimmed adaptor sequences, and removed low-quality bases from the raw paired-end reads: LEADING:3 Trailing:3 SlidingWINDOW:4:15 MINLEN:30. Clean readings were used for the analysis. HISAT2 (v2.1.0) aligned read pairs from each sample to the reference genome (e.g., Homo sapiens, GRCh38)^41,42^. FeatureCounts (v2.0.0) counted gene-mapped reads^43^. The “Trimmed Mean of M-values” (TMM) normalization (DEGseq [v1.40.0]^44^ without biological duplicate) and “Relative Log Expression” (RLE) normalization (DESeq2^45,46^ with biological duplicate) were used for gene expression. DEGseq (without biological replicates) and DESeq2 (with biological replicates) were used in R to analyze differentially expressed genes (DEGs) in the two situations^47–49^. GO and KEGG pathway^50,51^ clusterProfiler (v3.14.3) enriched DEGs^52^. DOSE^53^ maps disease ontology (DO) concepts to MeSH, ICD, NCI thesaurus, SNOMED, and OMIM. To discover enriched biological activities and activated pathways from MSigDB, gene set enrichment analysis (GSEA)^54^ was performed with 1000 permutations. MSigDB contains hallmark, positional, curated, motif, computational, GO, oncogenic, and immunological gene sets for GSEA software^55,56^. STRINGDB created a protein-protein interaction (PPI) network of DEGs. The Weighted Gene Co-expression Network Analysis (WGCNA) (v.1.69) in R was used to create a co-expression network based on expression pattern correlations^57,58^.

### Statistics and reproducibility

Data are presented as mean ± standard error of the mean (SEM). Pearson’s correlation coefficients were determined using a correlation analysis. Student’s *t*-test was used to evaluate significant differences between two groups, whereas one-way analysis of variance was used to compare more than two groups. A non-parametrical two-tailed Mann–Whitney U test or one-way analysis of variance (ANOVA) with the Tukey or Tukey–Kramer multiple comparison test was used. GraphPad Prism version 10 (GraphPad Software Inc., CA, USA) was used for statistical analyses. Statistical significance was set at *P* < 0.05. In the figure legends, the statistical methods applied to each figure are described, and the significance of each figure is mentioned.

## Supplementary information

Supplementary Information (new)

Description of Additional Supplementary Materials

Supplementary Data

Supplementary Data 2

Supplementary Data 3 NEW

## Data Availability

This study relied on publicly available data. Raw sequencing data files were uploaded to NCBI under the BioProject ID PRJNA1082498. The data supporting the findings of this study are available upon request from L.L.W. The source data behind the graphs in the main and Supplementary Figs. can be found in the supplementary Data file. Supplementary Data 2 and 3 comprise the bile acid report and bile acid raw data, respectively, which were acquired via liquid chromatography-mass spectrometry.
