# Author Correction: Nuclear factor interleukin 3 and metabolic dysfunction-associated fatty liver disease development

**DOI:** 10.1038/s42003-025-08902-2

**Published:** 2025-09-29

**Authors:** Yung-Ni Lin, Jia-Rou Hsu, Chih-Lin Wang, Yi-Chen Huang, Jzy-Yu Wang, Chun-Ying Wu, Li-Ling Wu

**Affiliations:** 1 Department and Institute of Physiology, College of Medicine, National Yang Ming Chiao Tung University, Taipei, Taiwan; 2 Institute of Clinical Medicine, National Yang Ming Chiao Tung University, Taipei, Taiwan; 3 Department of Family Medicine, National Cheng Kung University Hospital, Tainan, Taiwan; 4 Institute of Biomedical Informatics, National Yang Ming Chiao Tung University, Taipei, Taiwan; 5 Health Innovation Center, National Yang Ming Chiao Tung University, Taipei, Taiwan; 6 Microbiota Research Center, National Yang Ming Chiao Tung University, Taipei, Taiwan; 7 Division of Translational Research, Taipei Veterans General Hospital, Taipei, Taiwan; 8 Institute of Public Health, College of Medicine, National Yang Ming Chiao Tung University, Taipei, Taiwan; 9 Department of Public Health, China Medical University, Taichung, Taiwan

**Keywords:** Non-alcoholic fatty liver disease, Microbiome

Correction to: *Communications Biology* 10.1038/s42003-024-06565-z, published online 24 July 2024

In the published version on this article, there was an error in the company name “Genomics Bioscience Technology Co., Ltd.” and it should read “BIOTOOLS Co., Ltd.”. The original article has been corrected.

