# Supplementary material for: Nuclear factor interleukin 3 and metabolic dysfunction-associated fatty liver disease development
Source: Commun Biol. 2024 Jul 24;7:897. doi: 10.1038/s42003-024-06565-z (PMC11269659; doi:10.1038/s42003-024-06565-z)
Supplement: Supplementary file 1 — Supplementary Information (new) [file 42003_2024_6565_MOESM1_ESM.pdf]

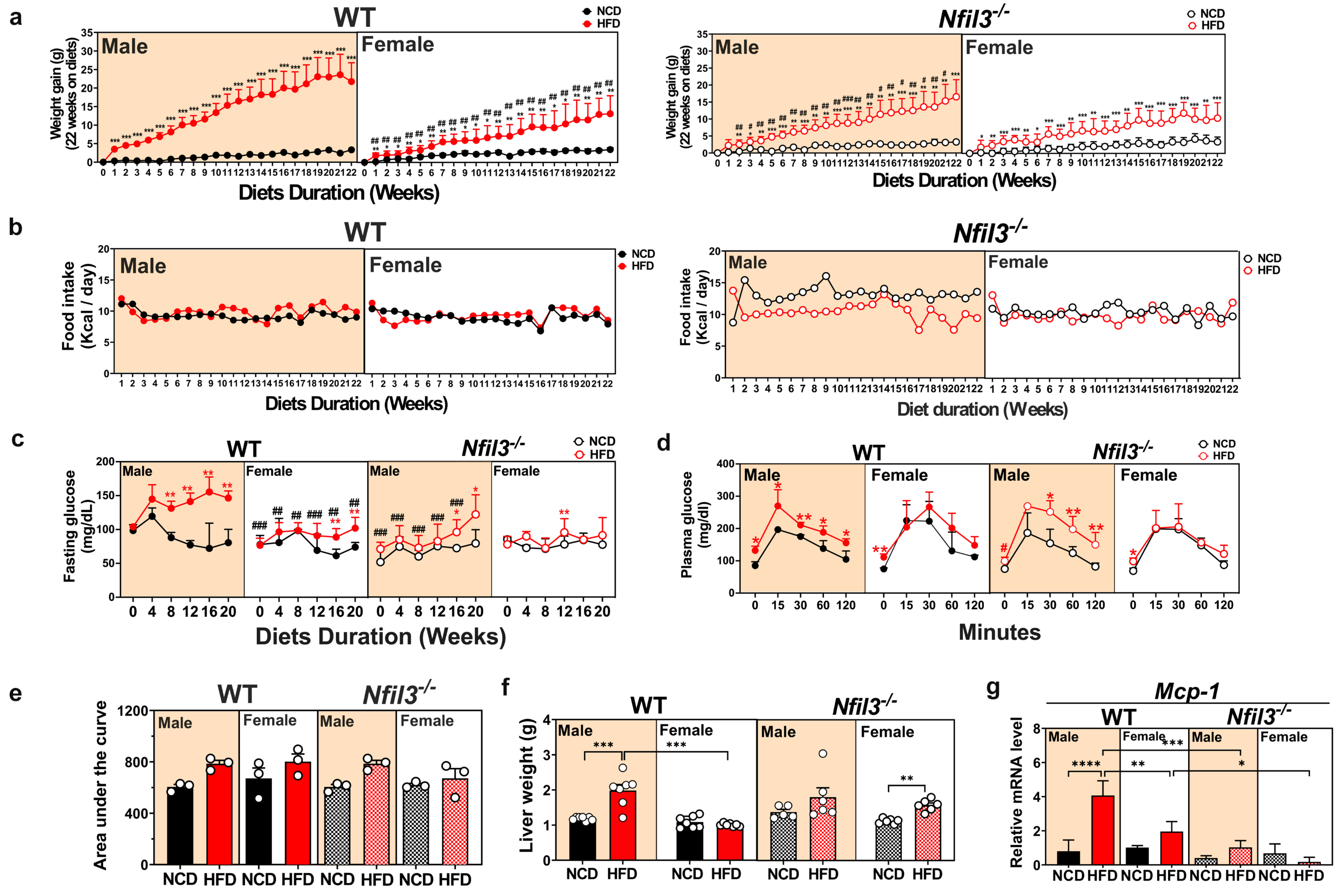

Supplemental figure 1

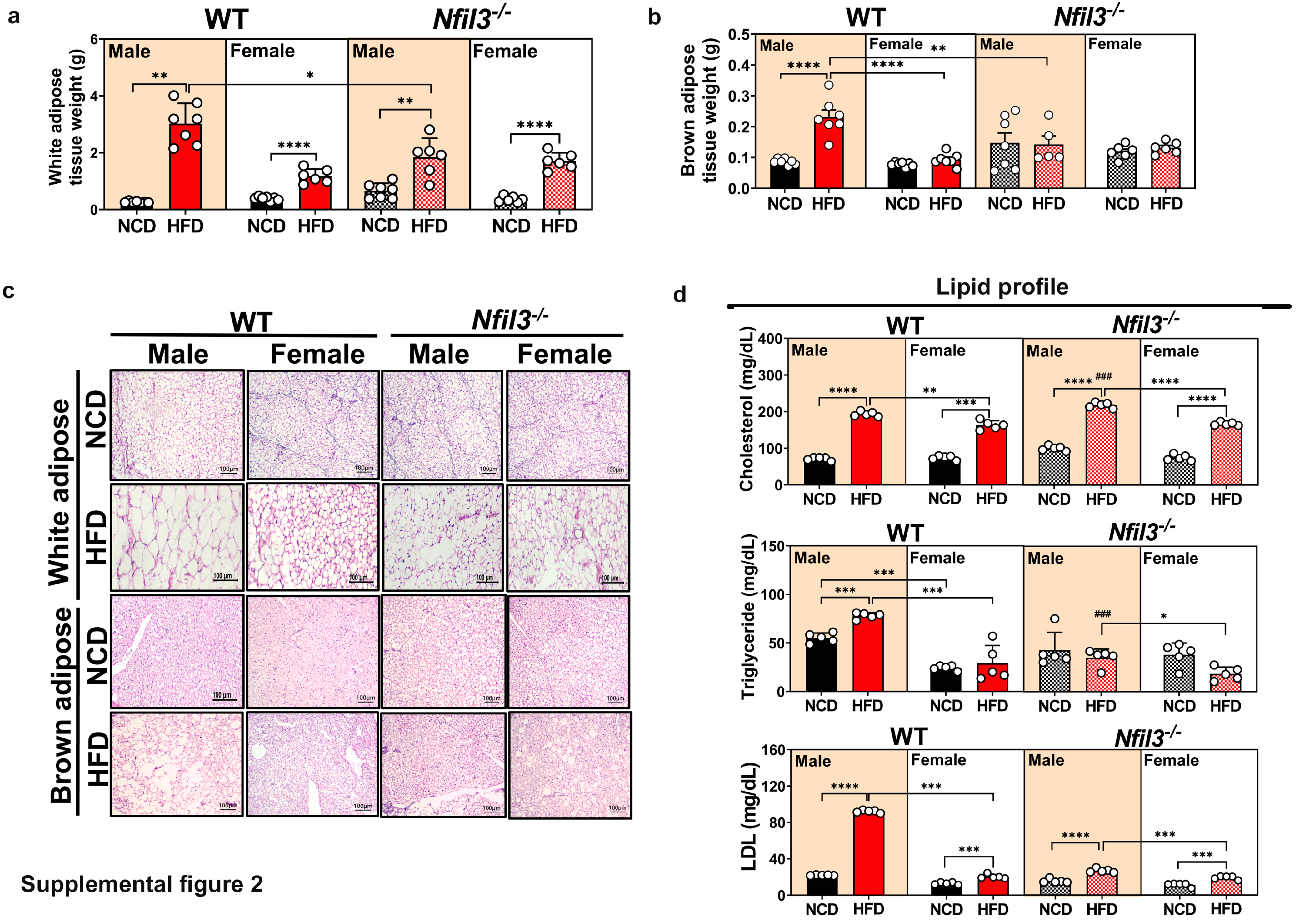

Supplemental figure 2

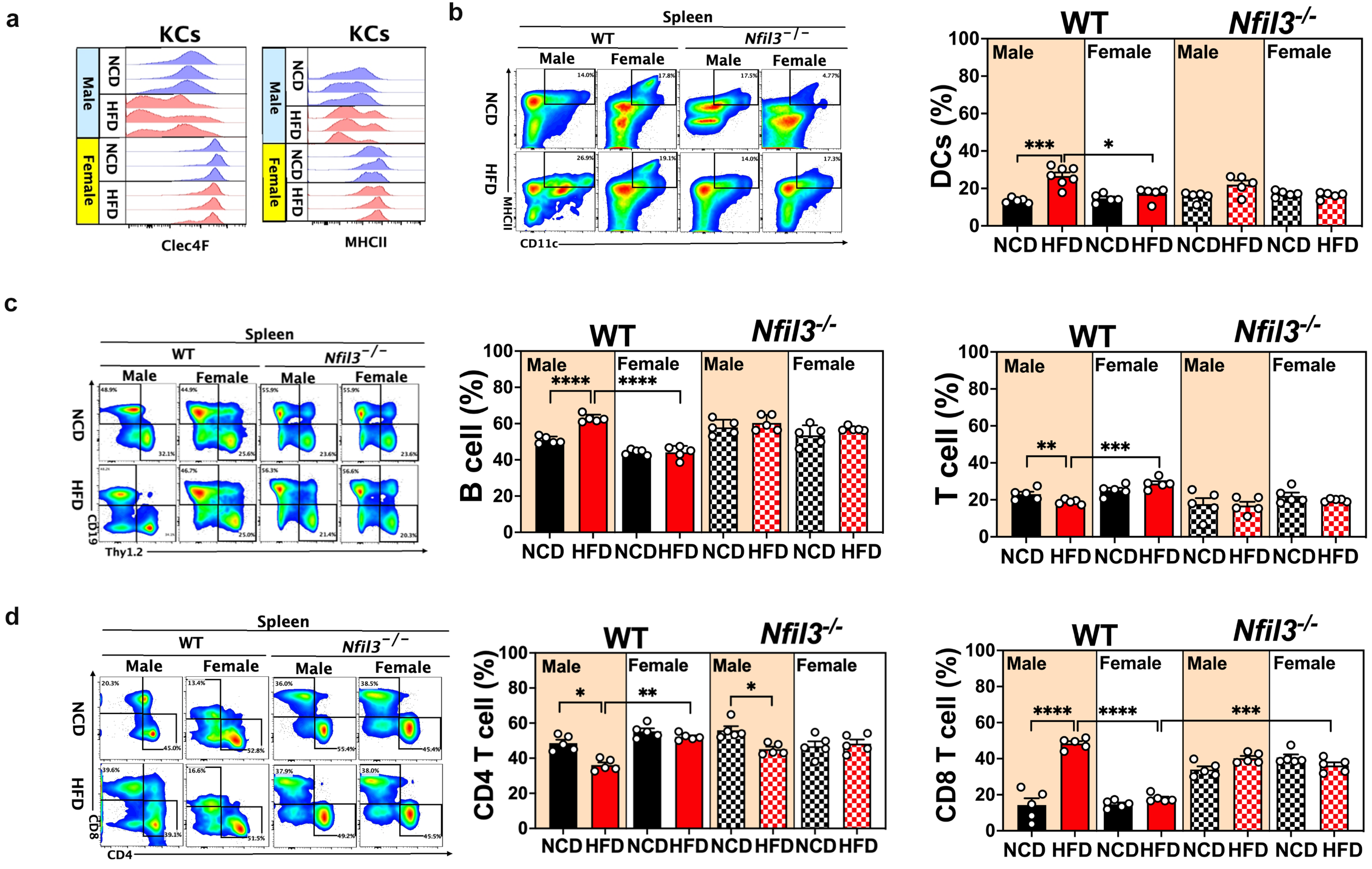

Supplemental figure 3

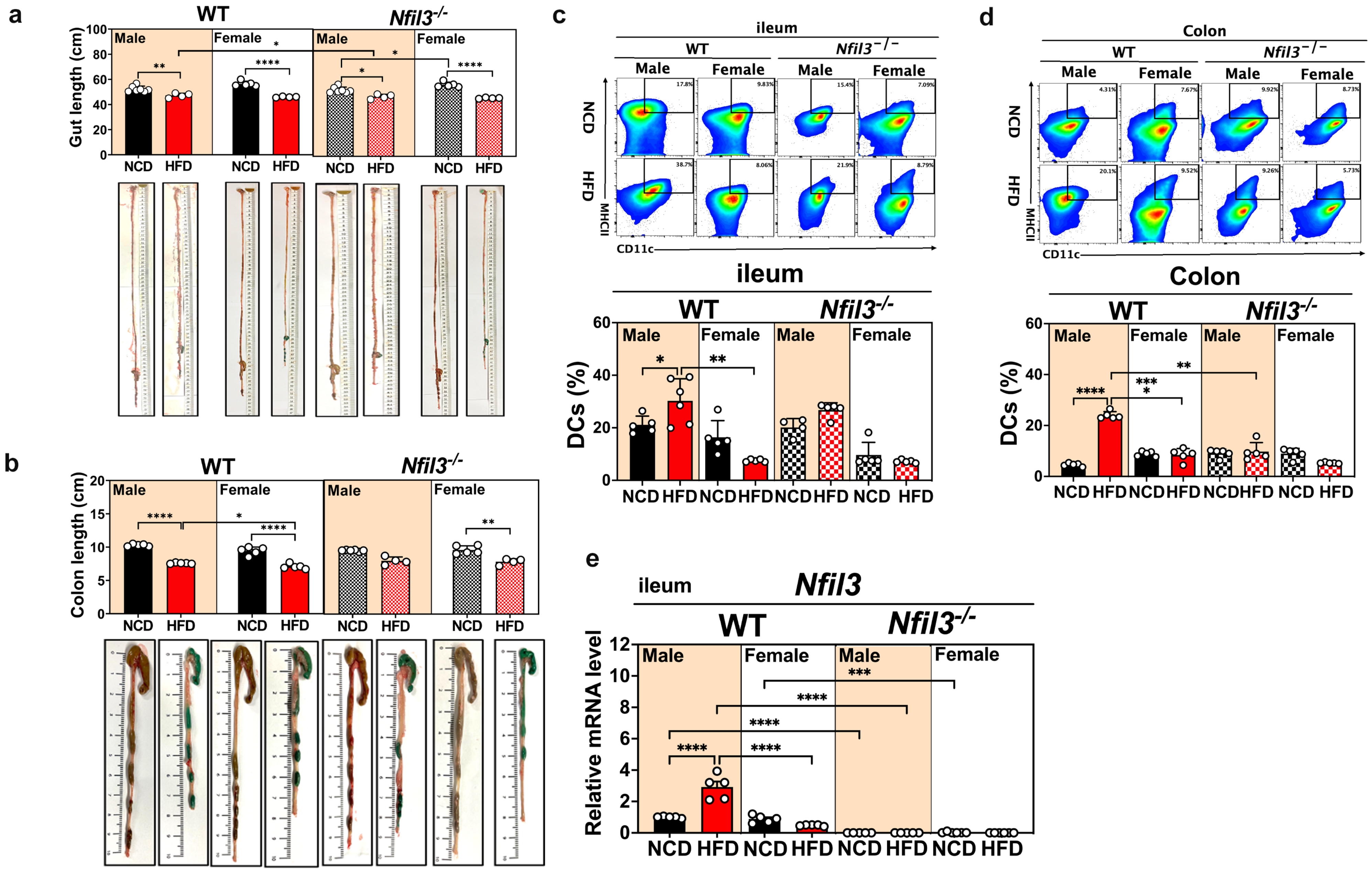

Supplemental figure 4

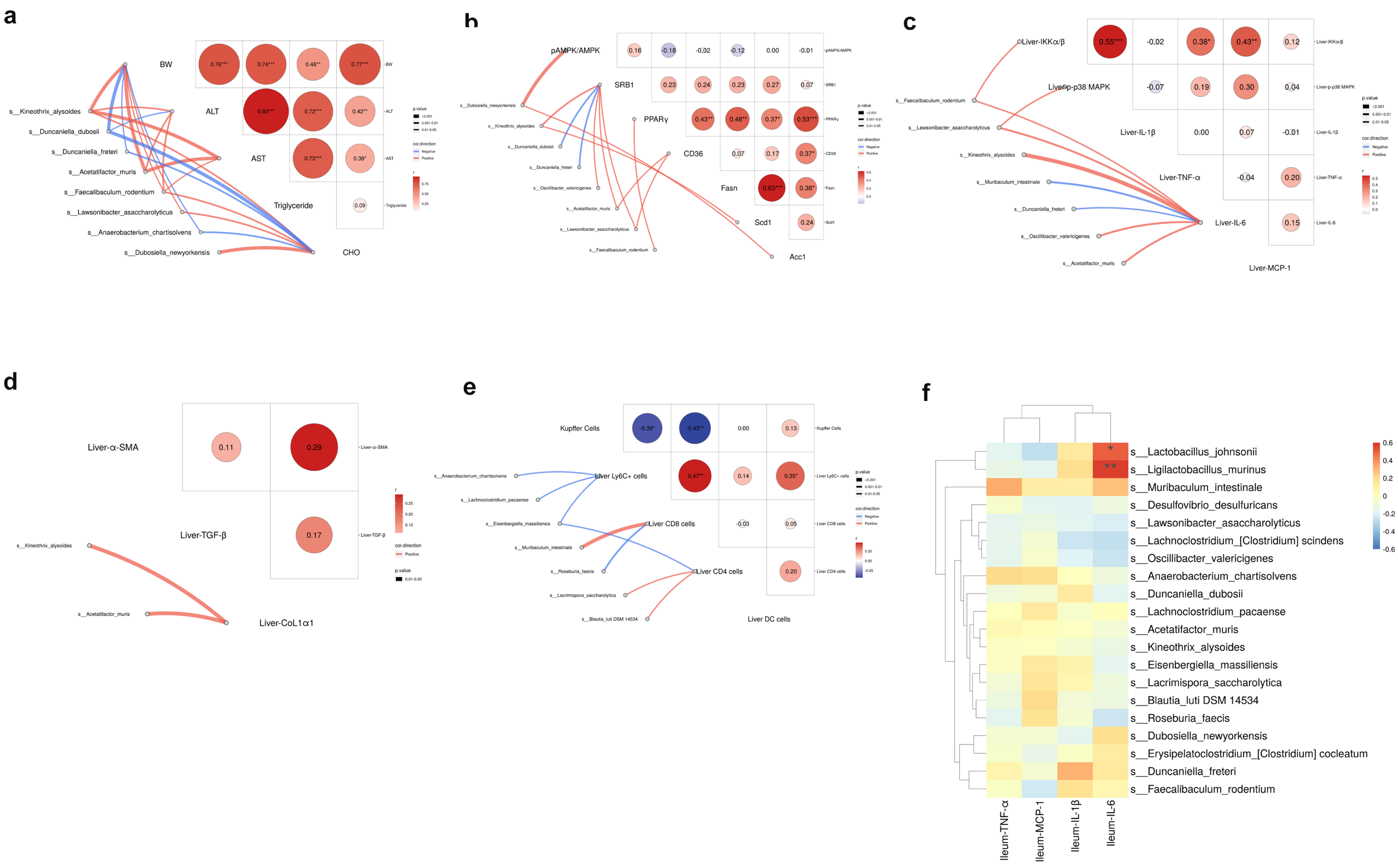

Supplemental figure 5

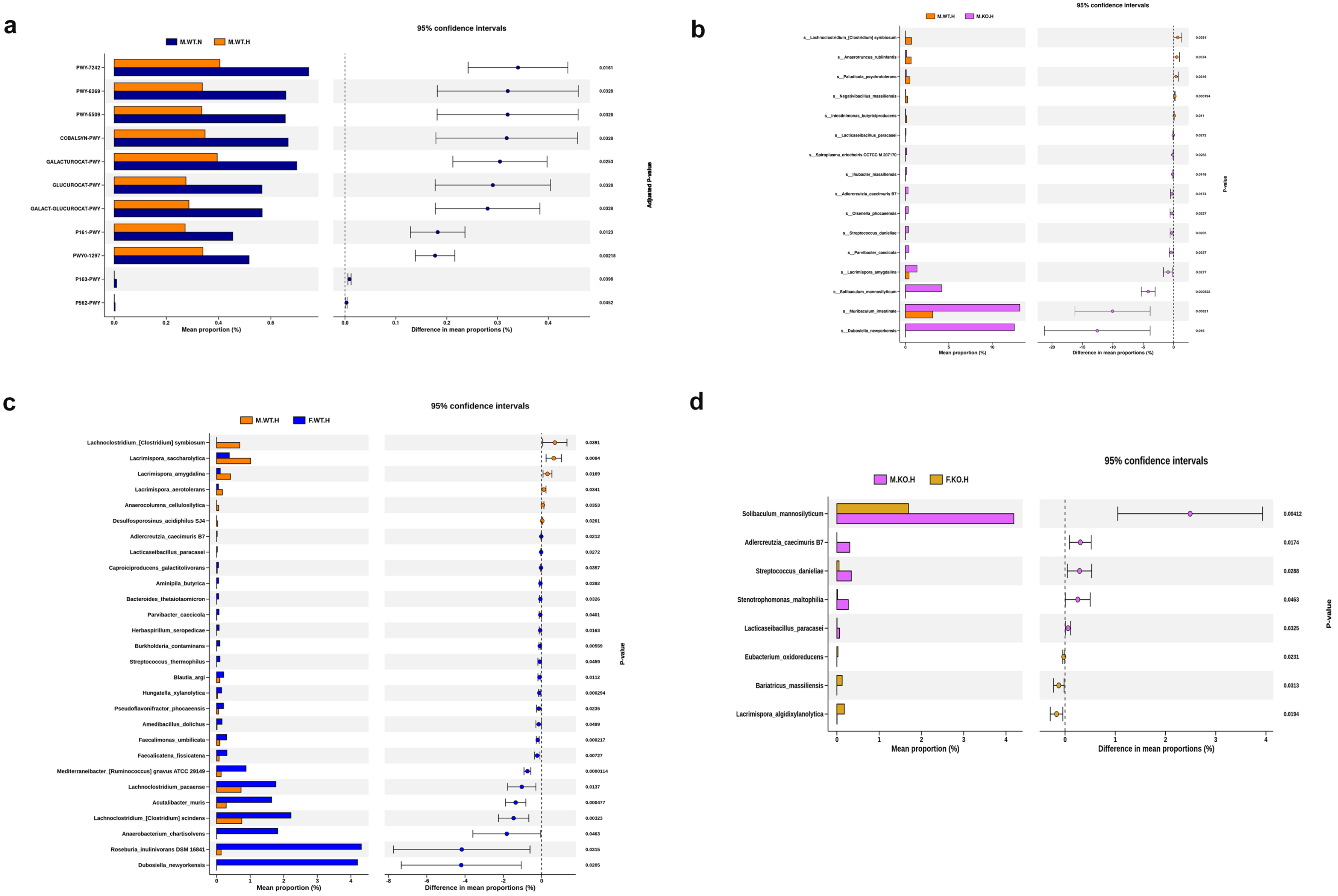

Supplemental figure 6

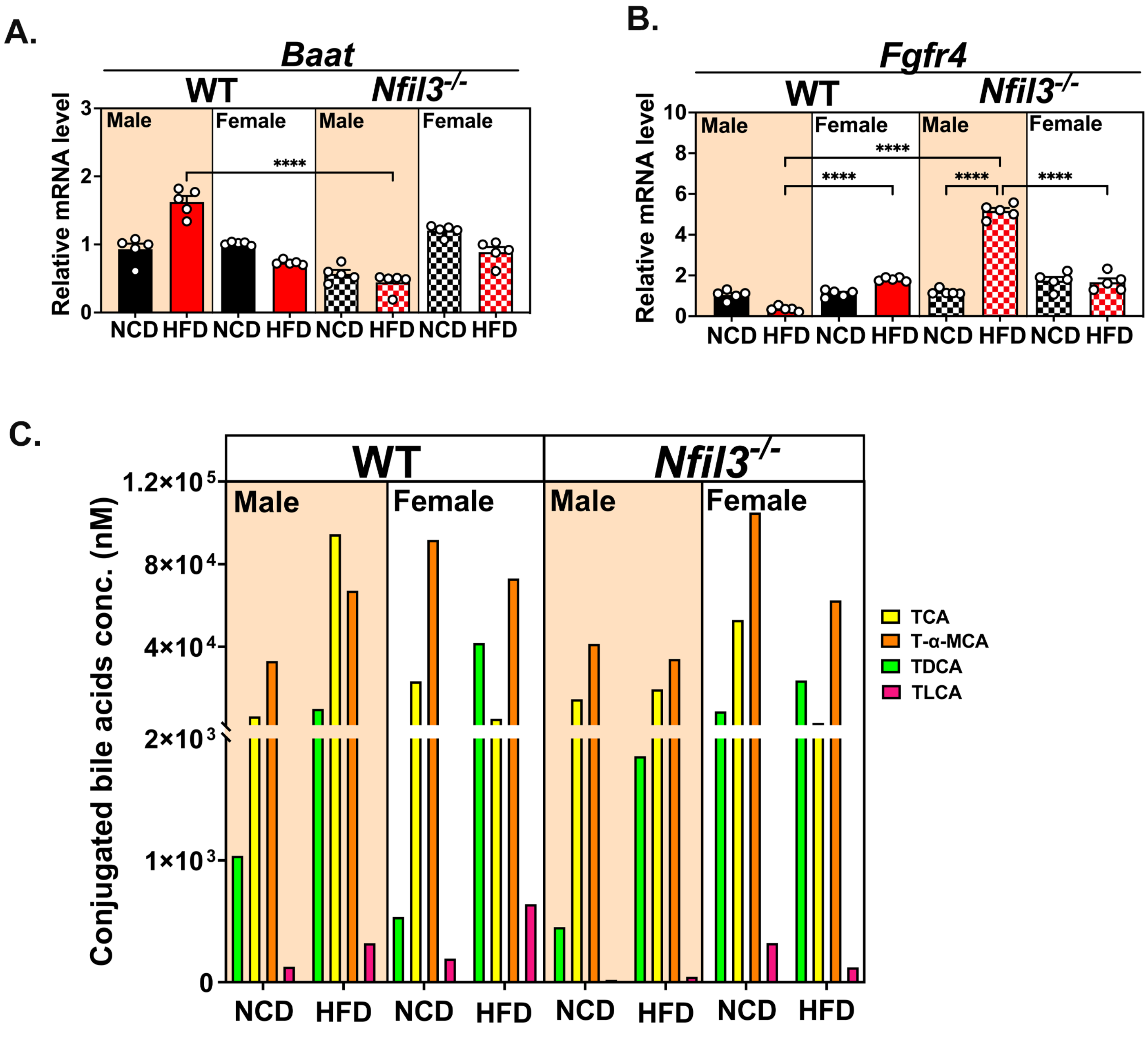

Supplemental figure 7

Supplementary Figure S8

Figure 1G

Figure 1G

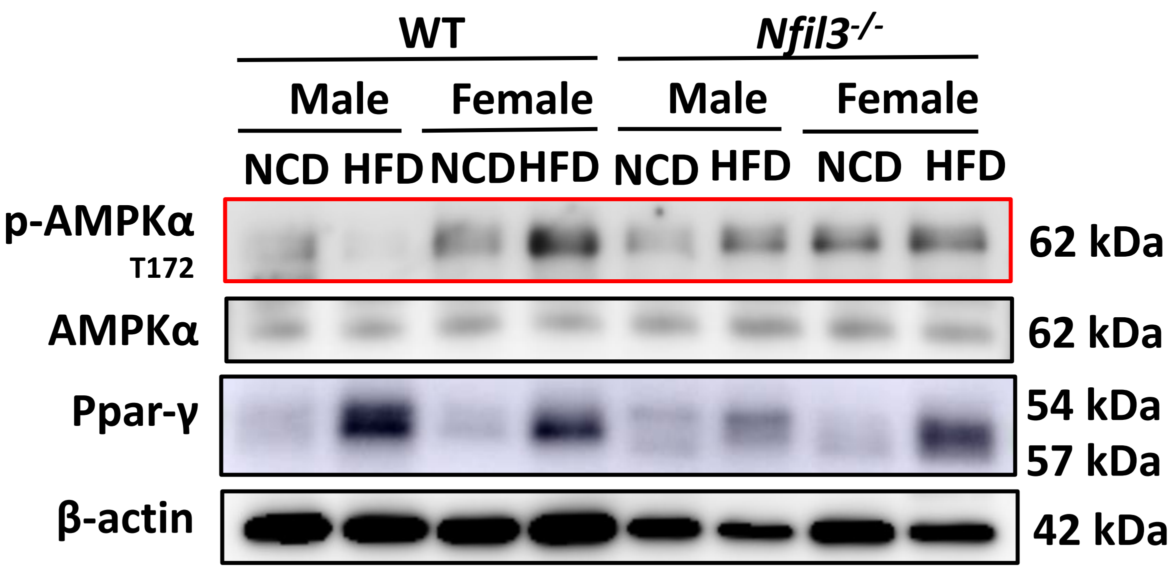

Present Data

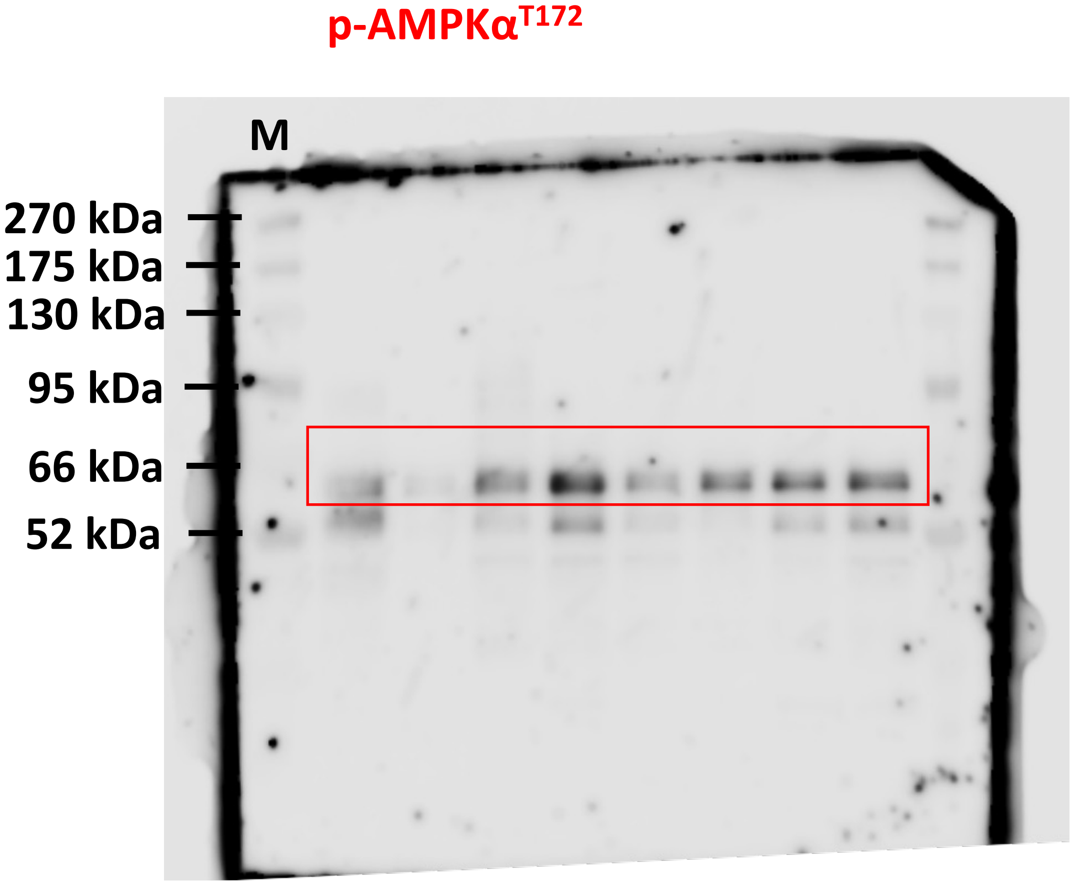

Figure 1G

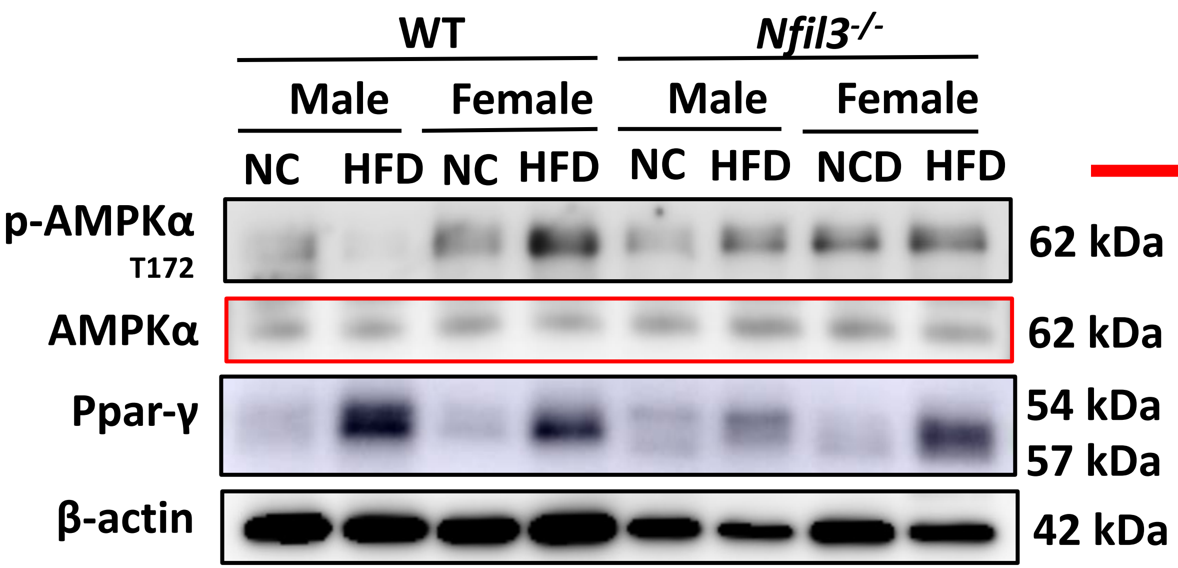

Raw Data

AMPKα

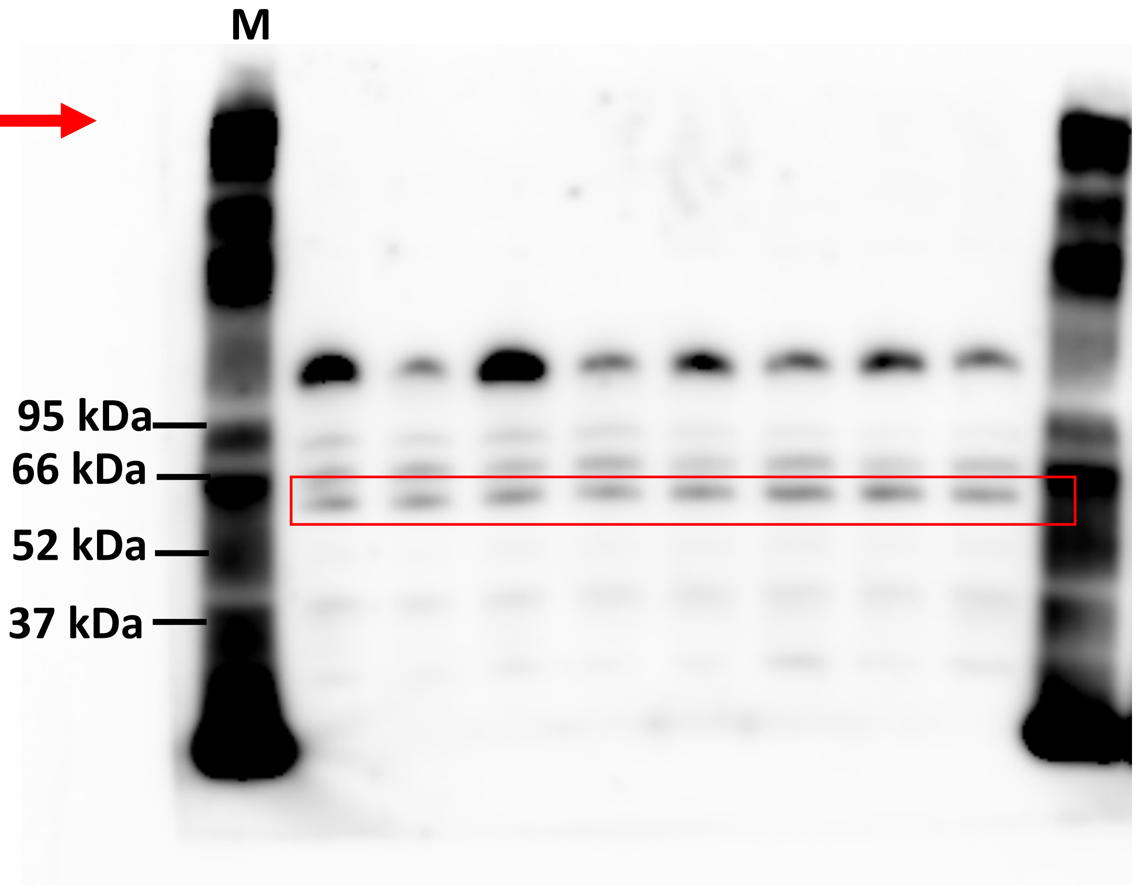

Figure 1G

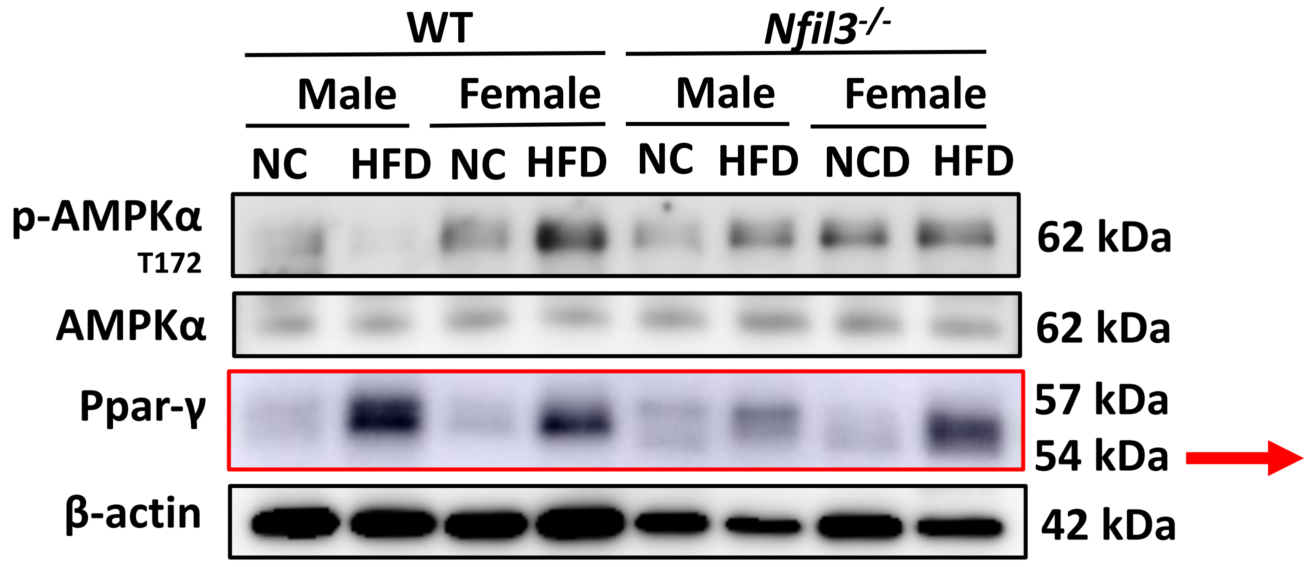

Raw Data

PPAR-γ

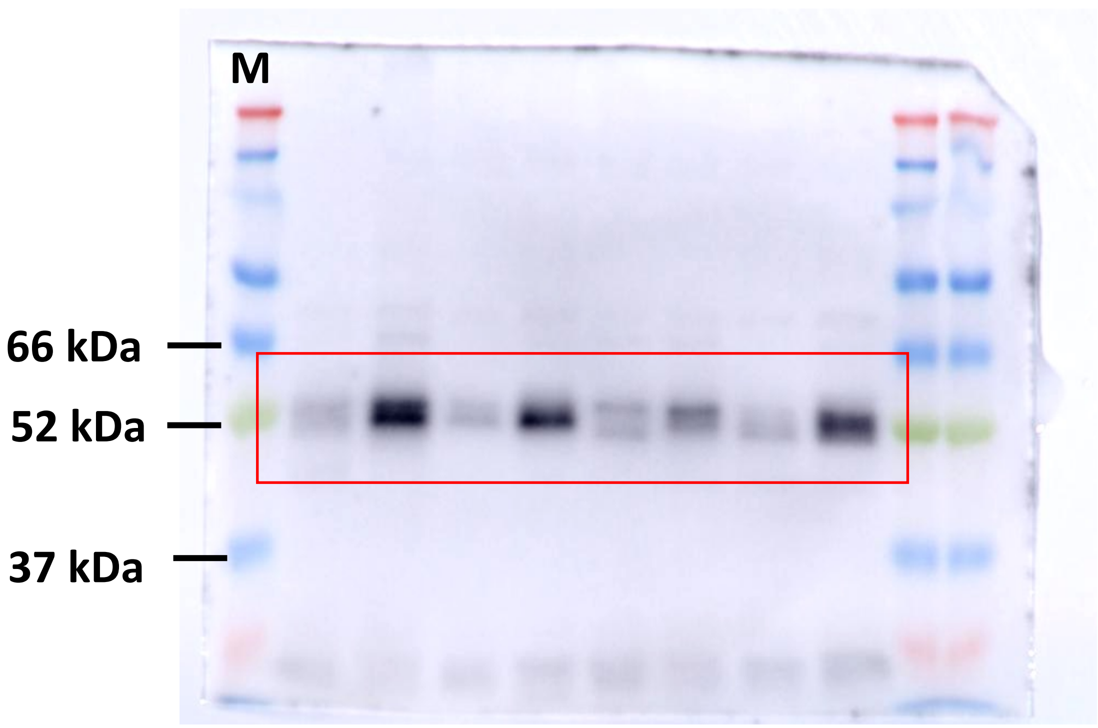

Figure 1G

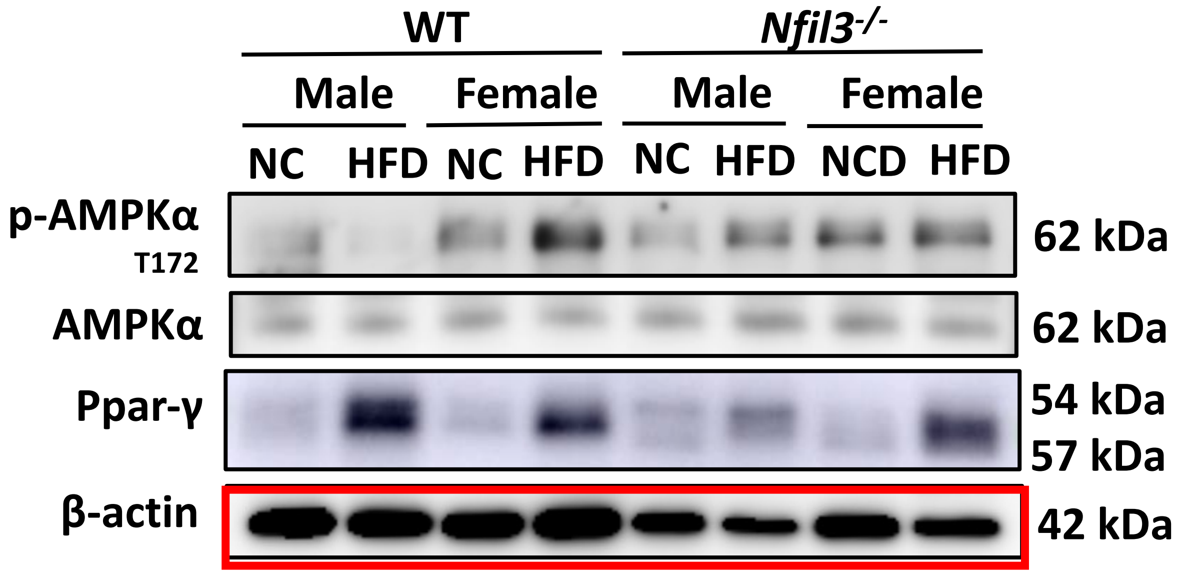

Raw Data

β-actin

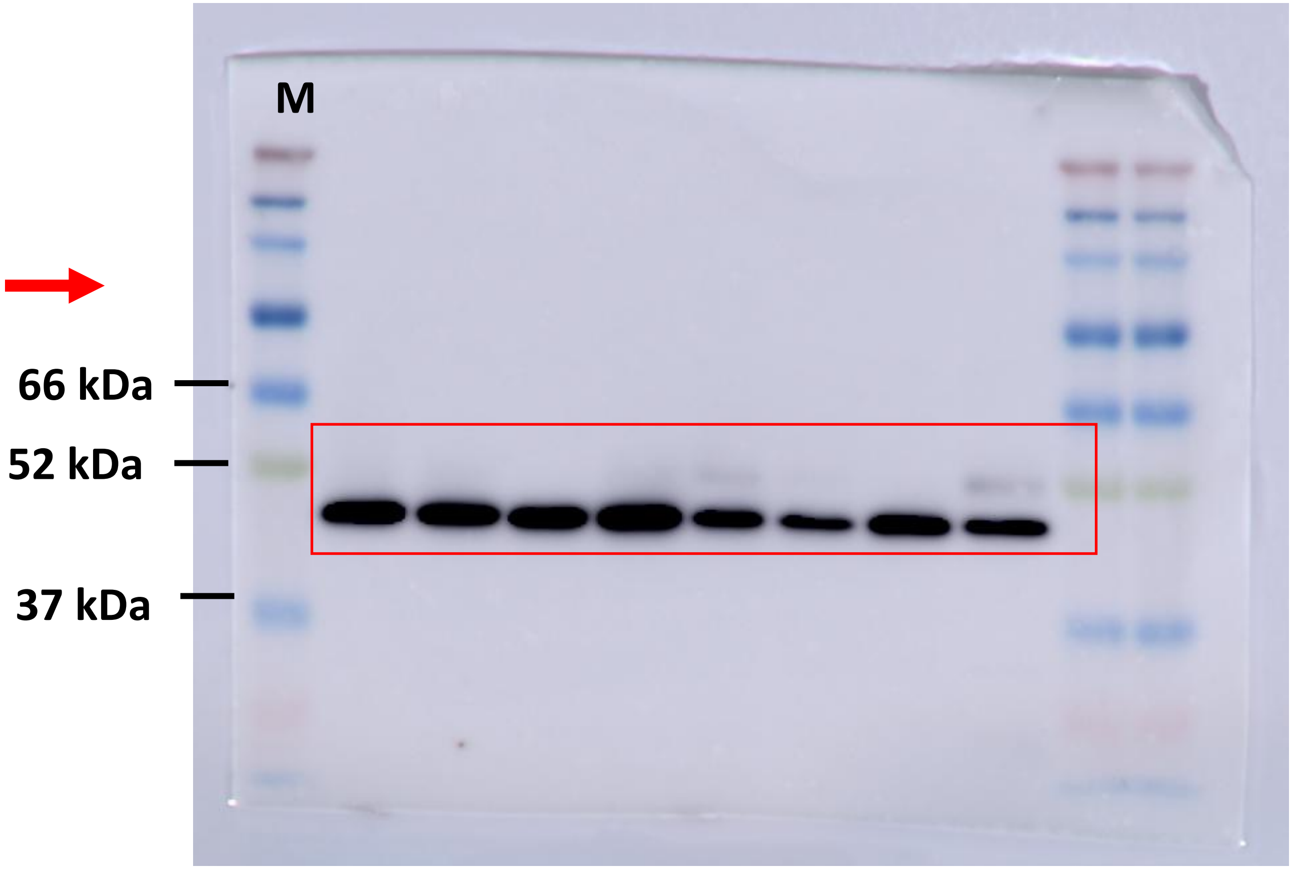

Figure 1G: p-AMPKα ^{T172} and AMPK α

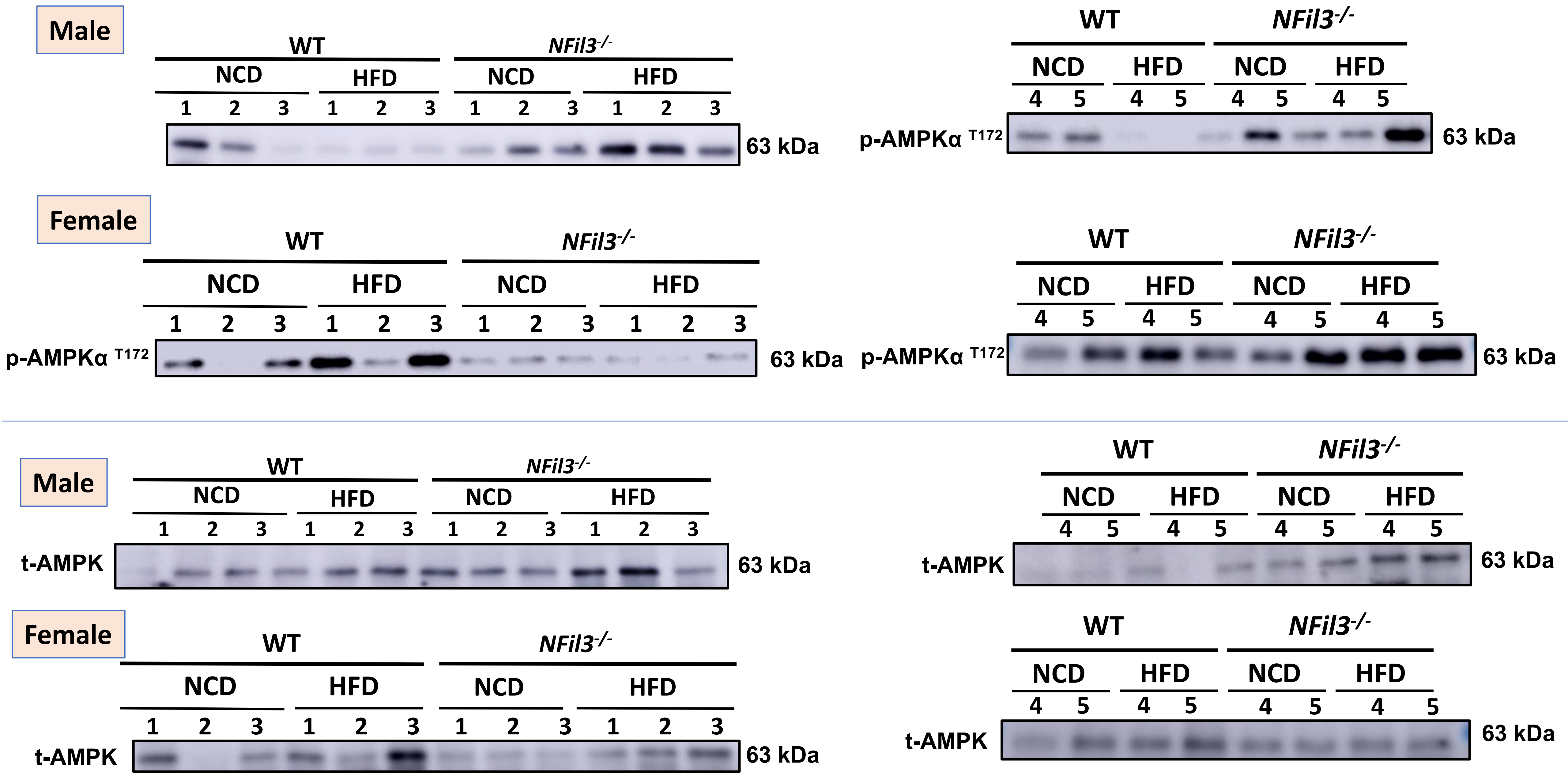

Figure 1G: Ppar-r

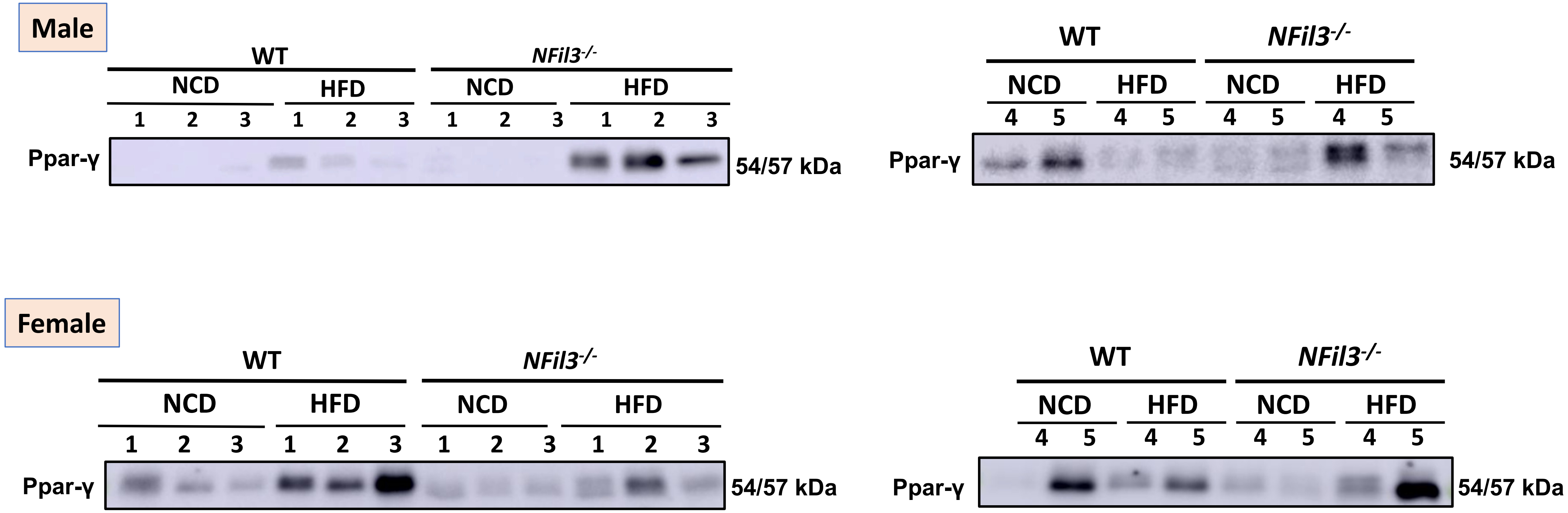

Figure 2D

Figure 2D

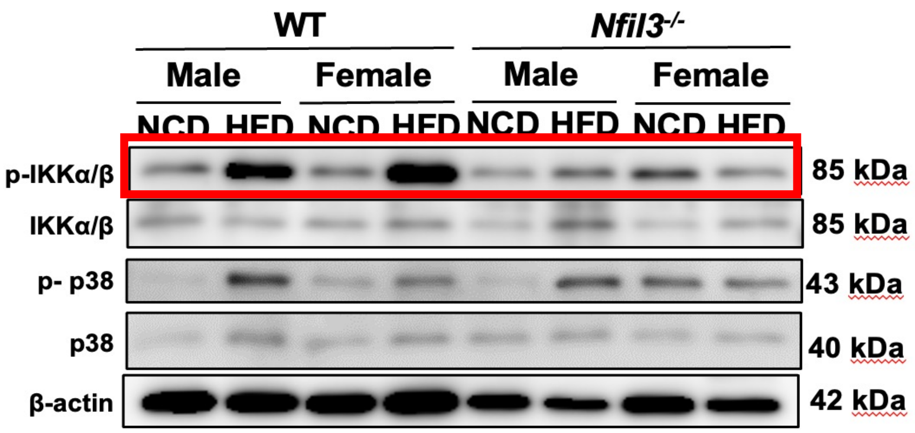

Raw Data

p-IKKα/β

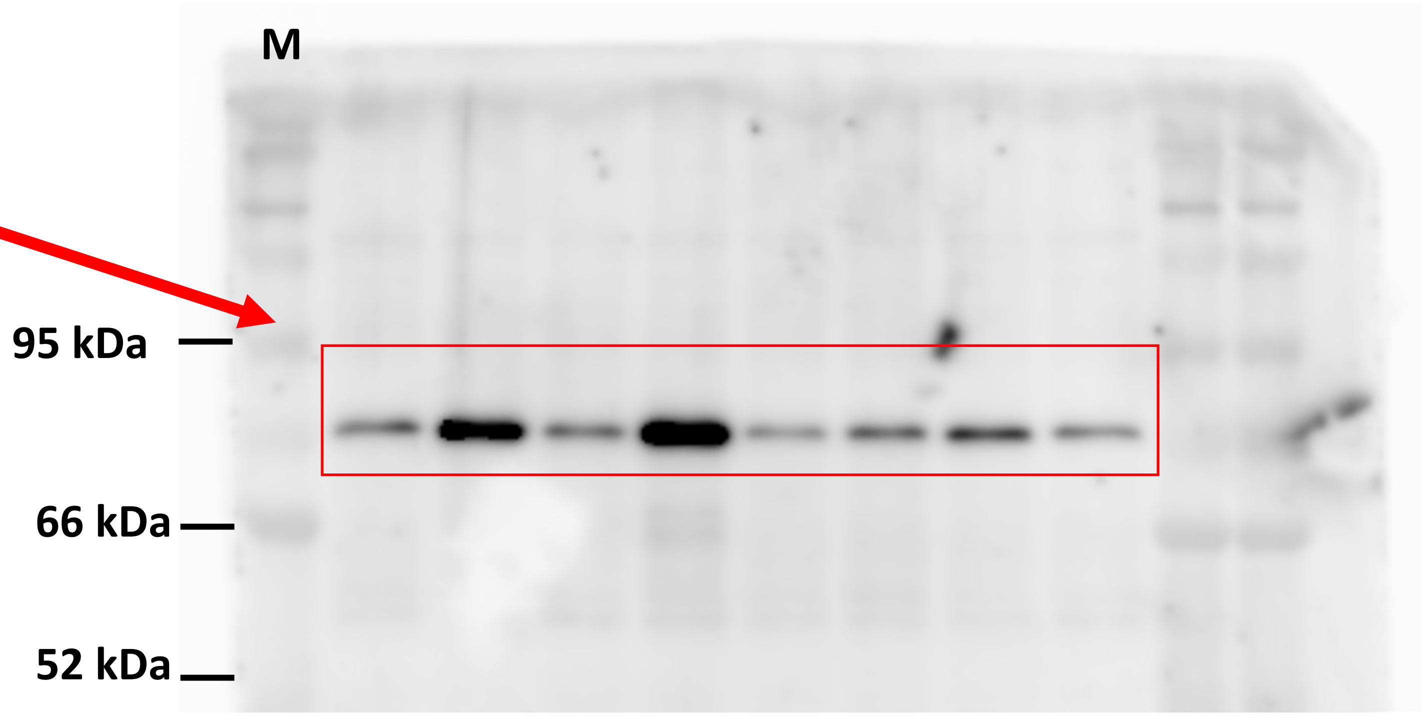

Figure 2D

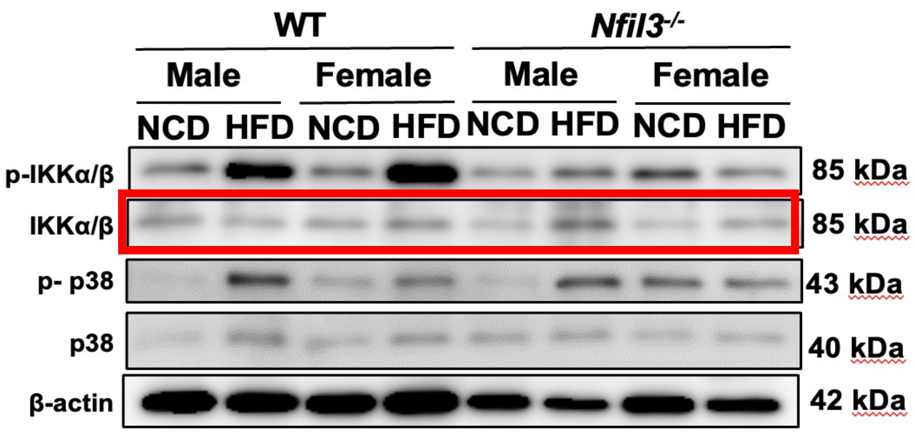

Raw Data

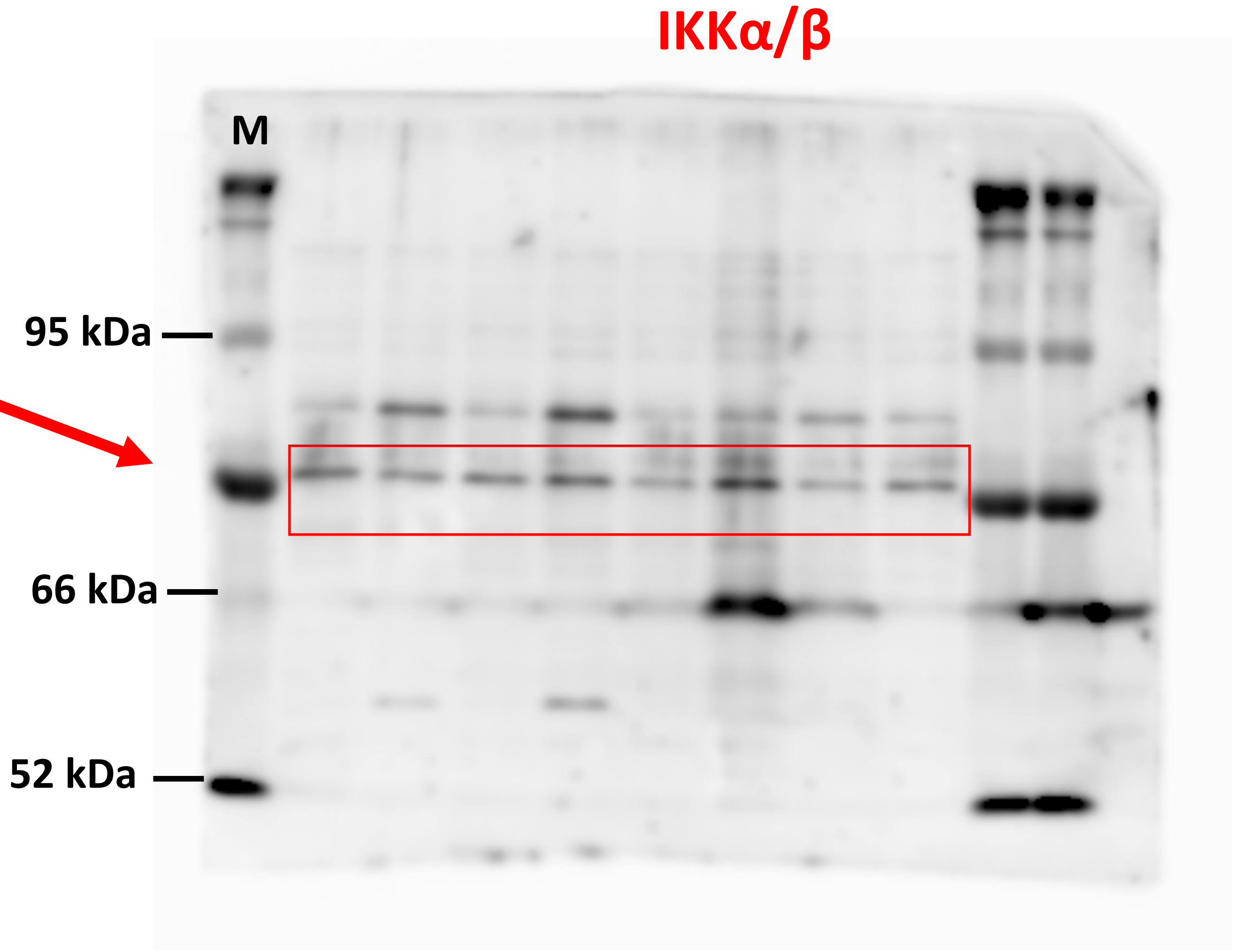

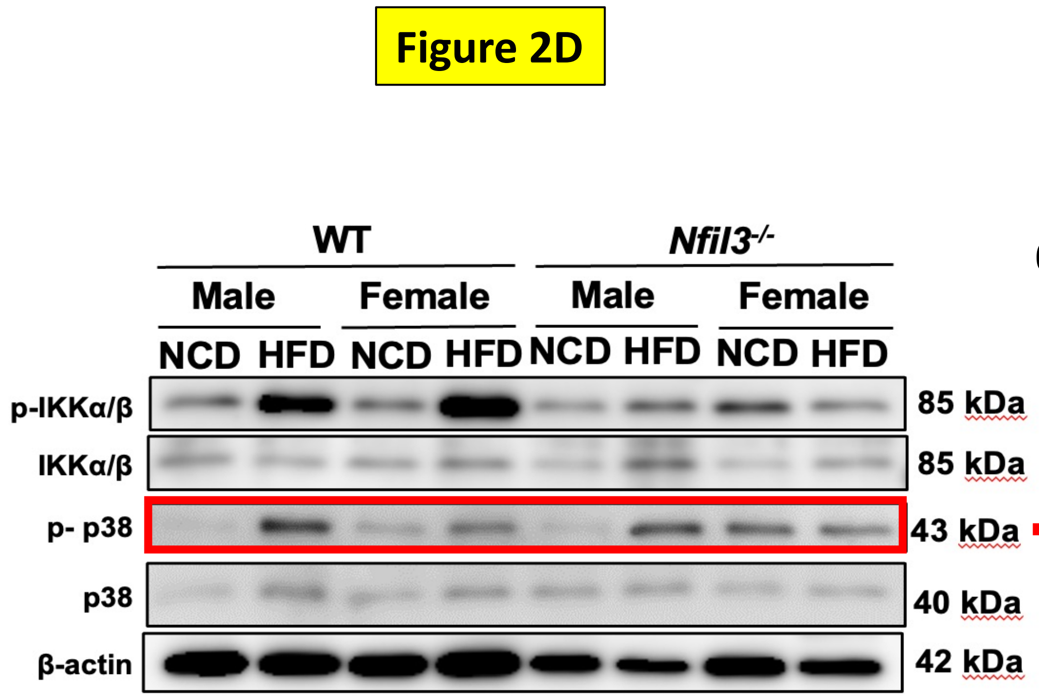
Figure 2D
WT
Nfil3-/-
Male
Female
Male
Female
NCD HFD NCD HFD NCD HFD NCD HFD
p-IKKα/β
IKKα/β
p- p38
p38
β-actin
85 kDa
85 kDa
43 kDa
40 kDa
42 kDa

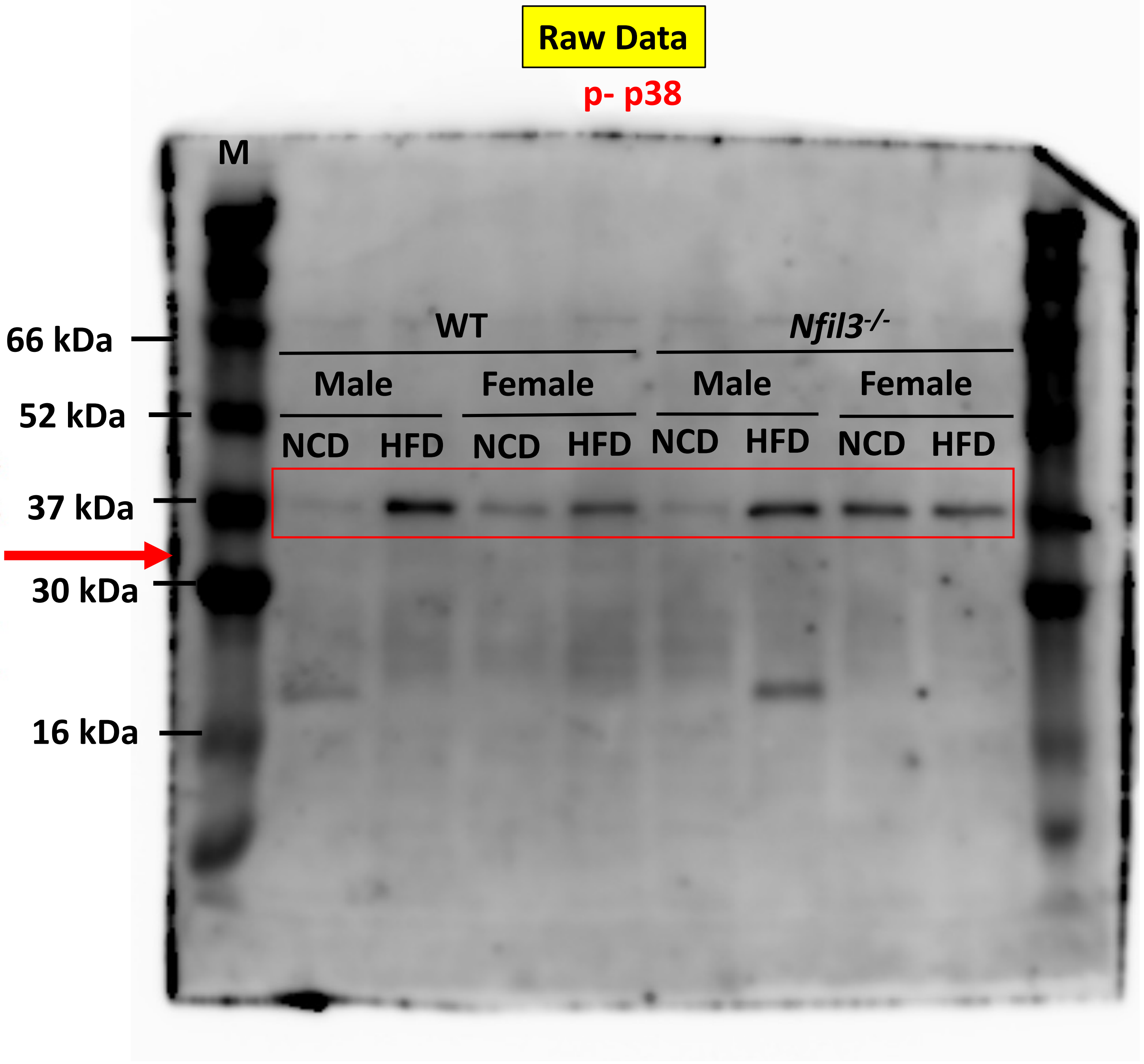
Raw Data
p- p38
M
66 kDa
52 kDa
37 kDa
30 kDa
16 kDa
WT
Nfil3-/-
Male
Female
Male
Female
NCD HFD NCD HFD NCD HFD NCD HFD

Figure 2D

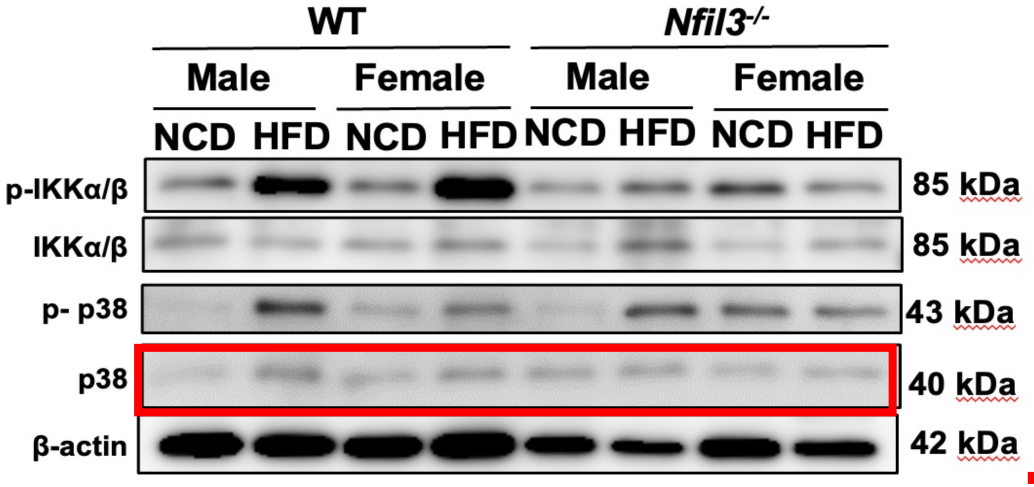

Raw Data

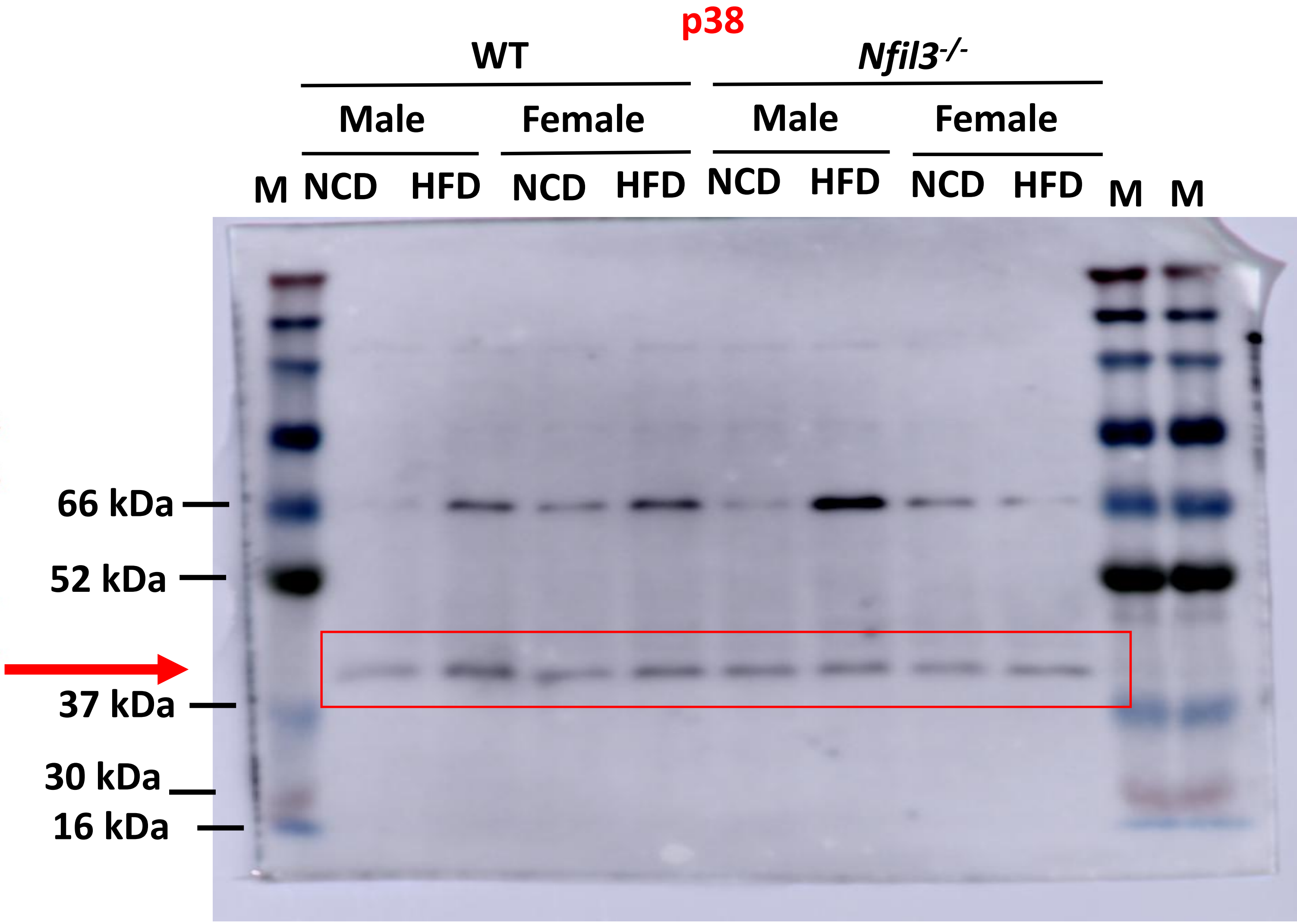

Figure 2D

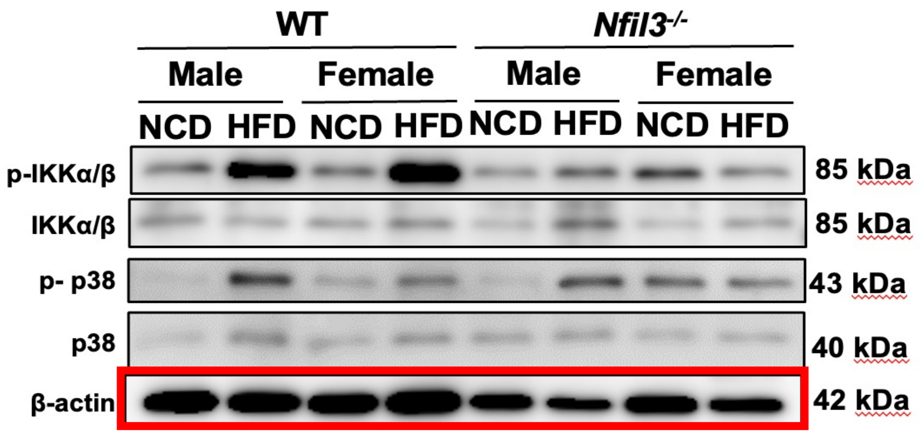

Raw Data

β-actin

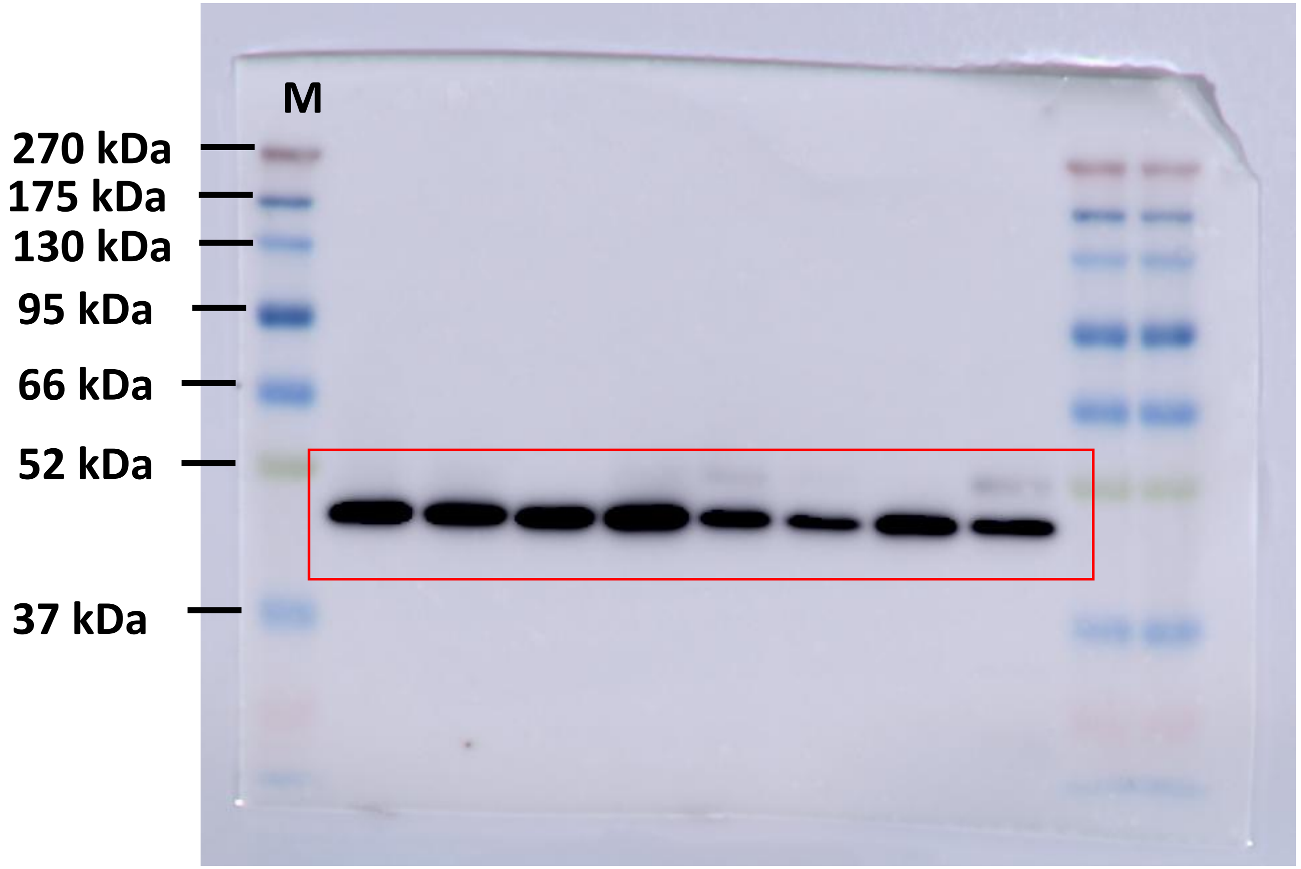

Figure 2D: p-IKKα/β

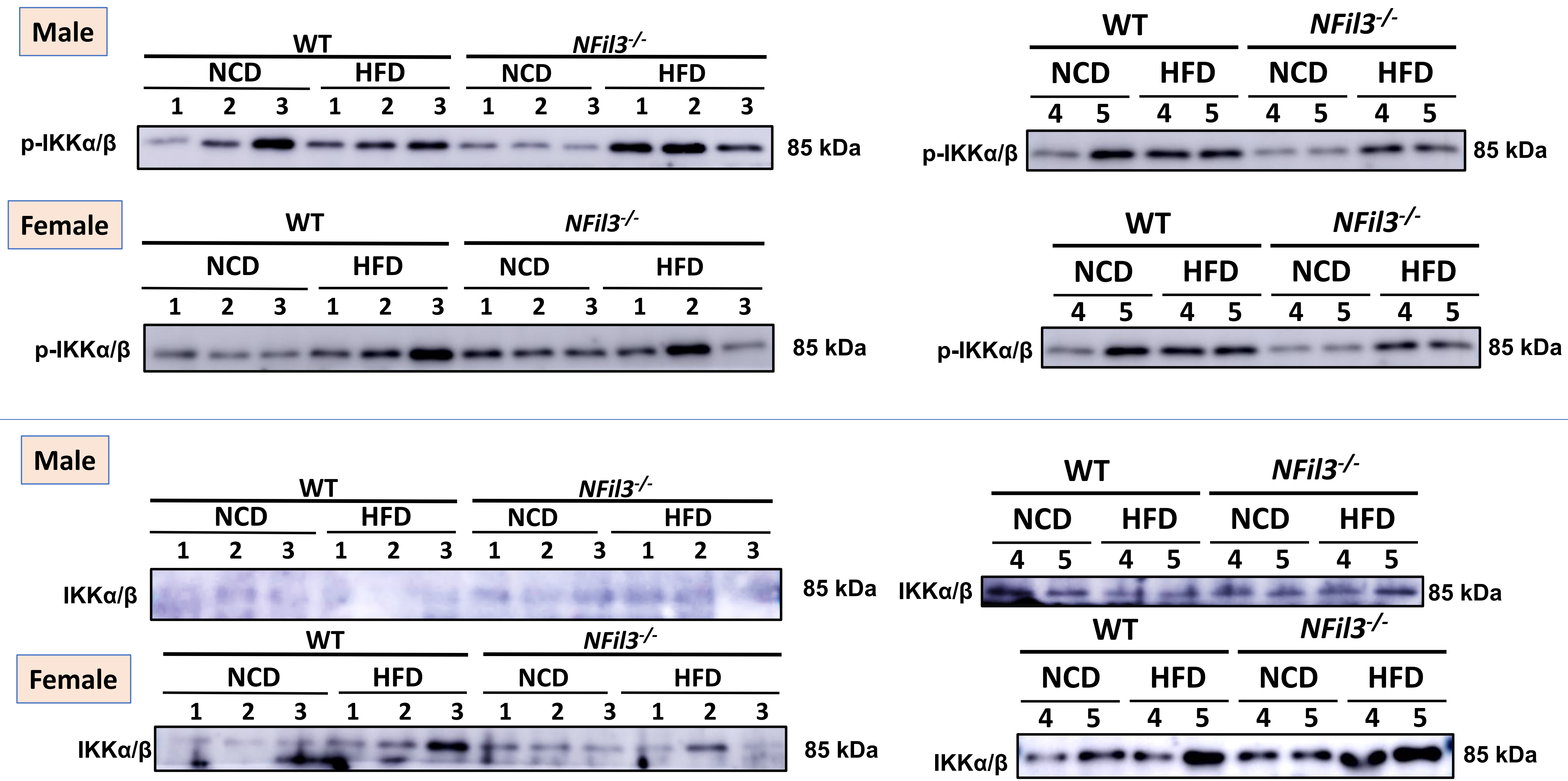

Figure 2D: p-p38 and p38

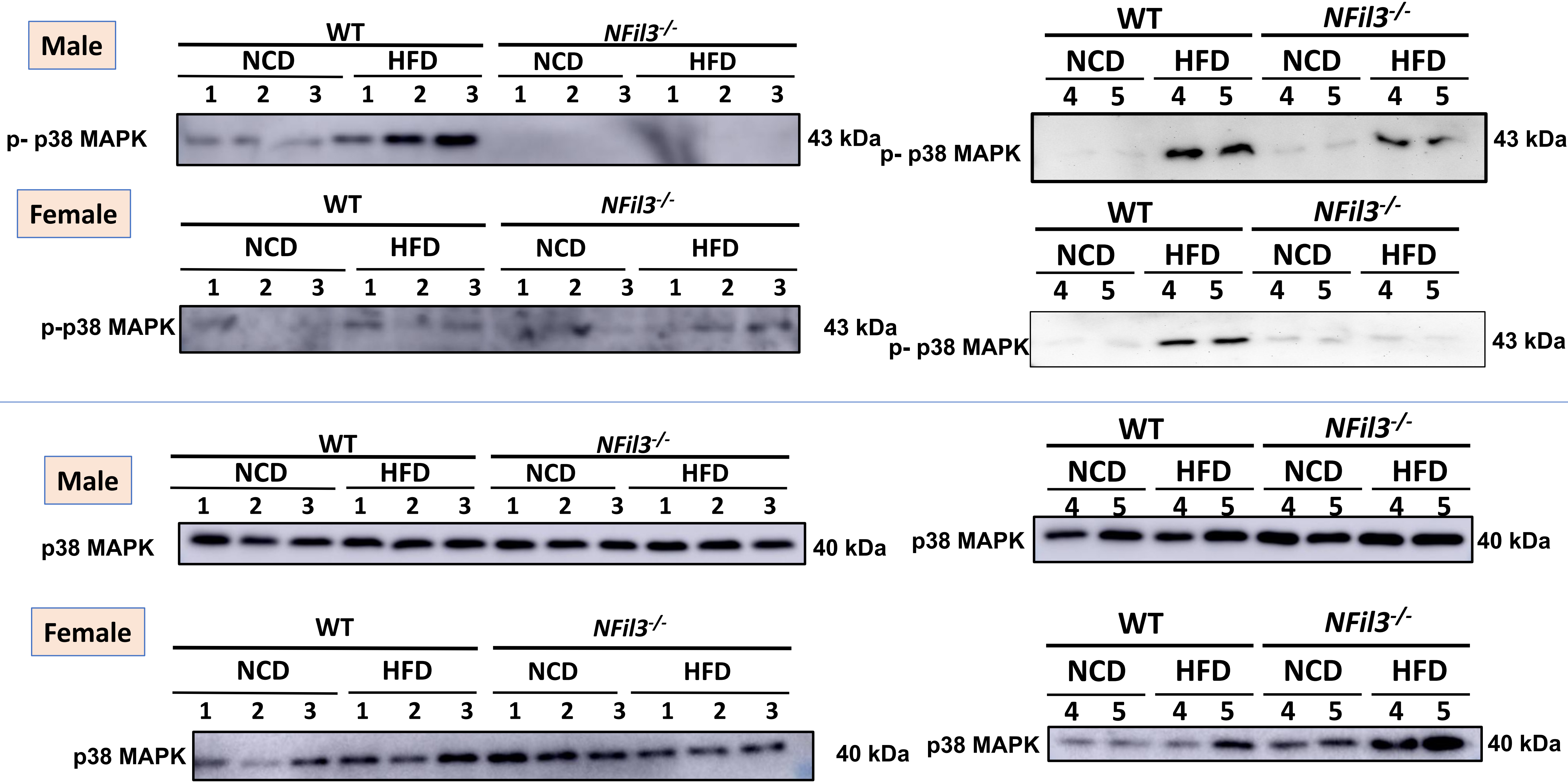

Figure 5I

Figure 5I

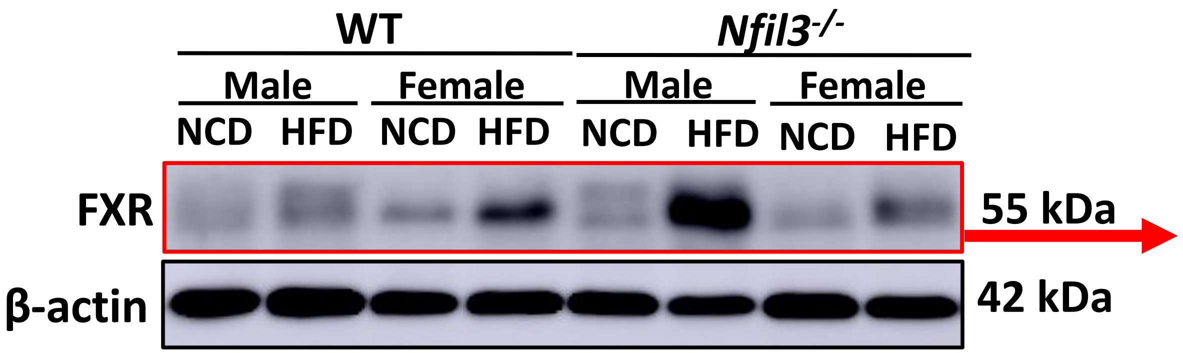

Raw Data

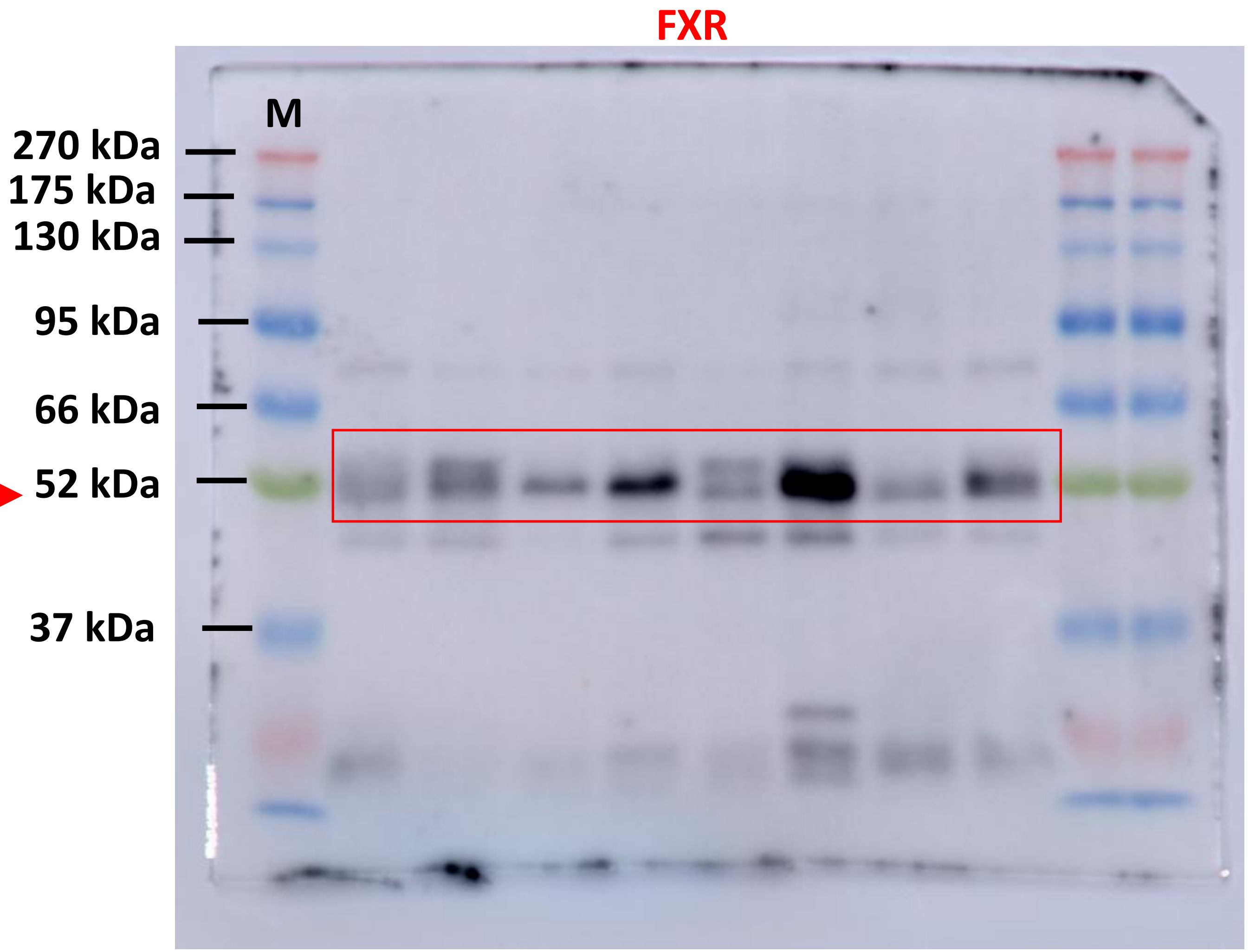

Figure 5I

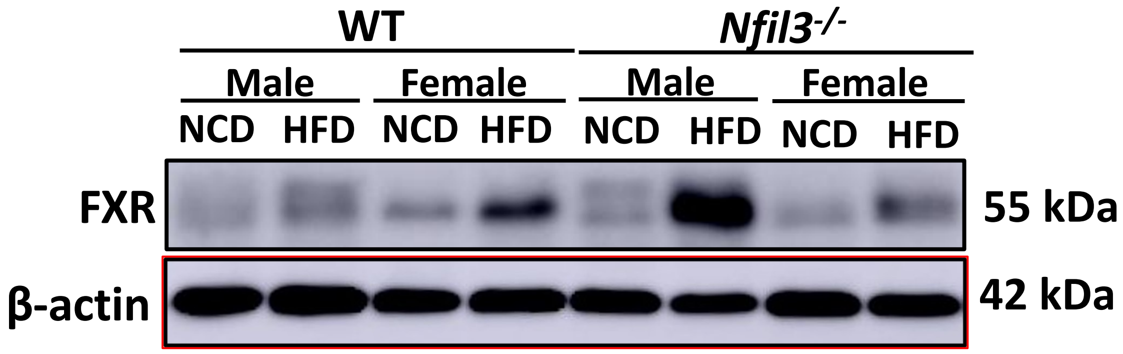

Raw Data

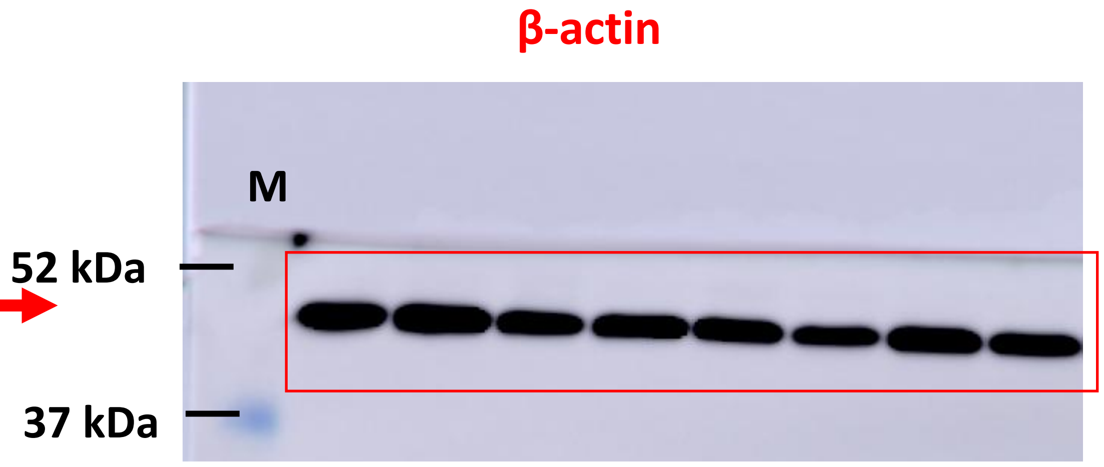

Figure 5I: FXR

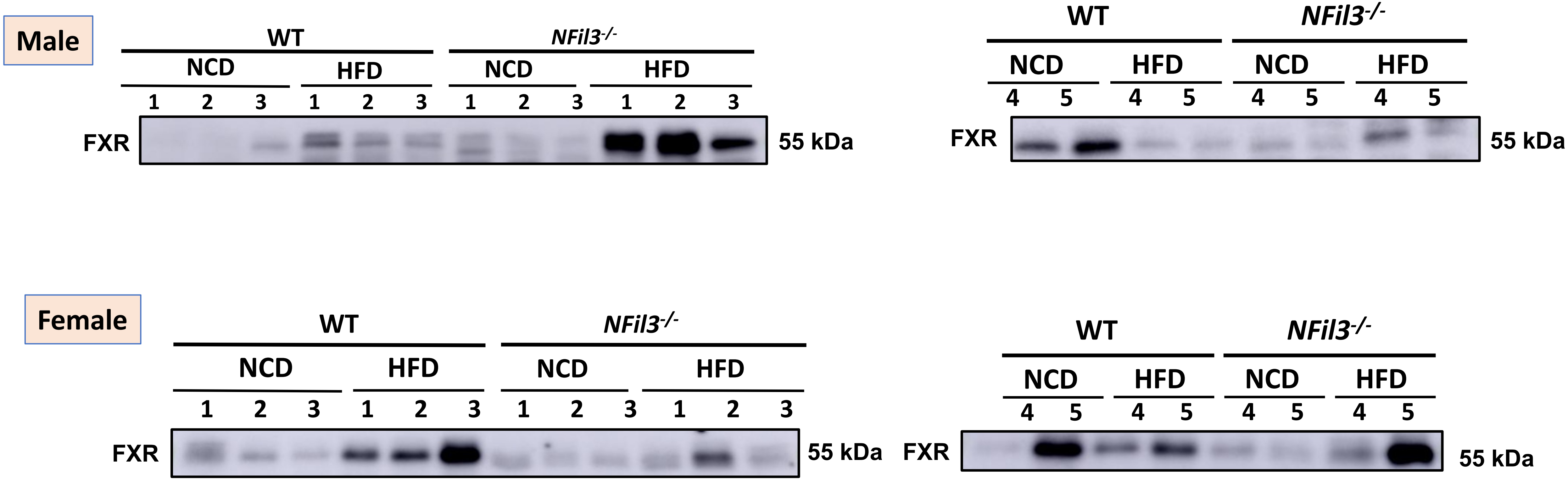

Table S1: Primer sequences for real-time polymerase chain expression used in the study

Primer	Sequence
Nfil3	Forward: CAGGACTACCAGACATCCAAGG<br>Reverse: AGGACACCTCTGACACATCGGA
Gapdh	Forward: TGTGTCCGTCGTGGATCTGA<br>Reverse: TTGCTGTTGAAGTCGCAGGAG
Tnf-α	Forward: ACGGCATGGATCTCAAAGAC<br>Reverse: GTGGGTGAGGAGCACGTAGT
Il-1β	Forward: TCTGAAGCAGCTATGGCAAC<br>Reverse: ATGAGTTGGGGACTCTCTGG
Il-6	Forward: TAGTCCTTCCTACCCCAATTTCC<br>Reverse: TTGGTCCTTAGCCACTCCTTCC
Mcp-1	Forward: CCCCAAGAAGGAATGGGTCC<br>Reverse: GTGCTGAAGACCTTAGGGCA
α-sma	Forward: GGCTCTGGGCTCTGTAAGG<br>Reverse: CTCTTGCTCTGGGCTTCATC
Col1a1	Forward: CCAAGGGTAACAGCGGTGAA<br>Reverse: CCTCGTTTTCCTTCTTCTCCG
Tgf-β1	Forward: GGTTCATGTCATGGATGGTGC<br>Reverse: TGACGTCACTGGAGTTGTACGG
Shp	Forward: TCTCTTCTTCCGCCCTATCA<br>Reverse: AAGGGCTTGCTGGACAGTTA
Fxr	Forward: CAAAATGACTCAGGAGGAGTACG<br>Reverse: GCCTCTCTGTCCTTGATGTATTG
Bacs	Forward: GTG GGT GAA ATC CTG CGG TA<br>Reverse: GAC CAA AGC GTT GCT GGA AG
Baat	Forward: GGAAACCTGTTAGTTCTCAGGC<br>Reverse: GTGGACCCCCATATAGTCTCC
Ntcp	Forward: ATGACCACCTGCTCCAGCTT<br>Reverse: GCCTTTGTAGGGCACCTTGT
Oatp	Forward: CAGTCTTACGAGTGTGCTCCAGAT<br>Reverse: ATGAGGAATACTGCCTCTGAAGTG
Ostα	Forward: TGTTCCAGGTGCTTGTCATCC<br>Reverse: CCACTGTTAGCCAAGATGGAGAA
Ostβ	Forward: GATGCGGCTCCTTGGAATTA<br>Reverse: GGAGGAACATGCTTGTCATGAC

Asbt	Forward: CTT CTC CCC CGA GGA TCT CA<br>Reverse: TGA TGG CCT GGA GTC CAT TTC
Fgfr4	Forward: ATT CCT GGC TCT TCG GCC C<br>Reverse: CAG ACT TCC CAC TGA CCA CC
Fgf15	Forward: ACG GCA AGA TAT ACG GGC TG<br>Reverse: GGC TTG GCC TGG ATG AAG AT
Cyp7a1	Forward: CCT CTG GGC ATC TCA AGC AA<br>Reverse: AAT GGC ATT CCC TCC AGA GC
Cd36	Forward: CCAAGCTATTGCGACATGATT<br>Reverse: CCGAACCACAGCGTAGATAGACC
Fasn	Forward: TTGGCCCAGAACTCCTGTAG<br>Reverse: CTCGCTTGTCGTCTGCCT
Acc1	Forward: GAAGCCACAGTGAAATCTCG<br>Reverse: GATGGTTTGGCCTTTCACAT
Scd1	Forward: GCCGAGCCTTGTAAGTTCTG<br>Reverse: CCTCCTGCAAGCTCTACACC
Occludin	Forward: ACGGACCCTGACCACTATGA<br>Reverse: TCAGCAGCAGCCATGTACTC
Zo-1	Forward: GGGGCCTACACTGATCAAGA<br>Reverse: TGGAGATGAGGCTTCTGCTT

Table S2 List of antibodies used for western blot analysis

No.	Antibody	Manufacturer	Catalog No.	Dilution
1	p-AMPKα-T172	Cell signaling	2535	1:1000
2	AMPKα	Cell signaling	5831	1:1000
3	Ppar-r	Santa Cruz	sc-7273	1:1000
4	p-IKKα/β	Santa Cruz	sc-21661	1:1000
5	P-p38 MAPK	Cell signaling	4511	1:1000
6	P38-MAPK	Cell signaling	8690	1:1000
7	FXR	Cell signaling	72105	1:1000
