# Supplementary material for: Nuclear factor interleukin 3 and metabolic dysfunction-associated fatty liver disease development
Source: Commun Biol. 2024 Jul 24;7:897. doi: 10.1038/s42003-024-06565-z (PMC11269659; doi:10.1038/s42003-024-06565-z)
Supplement: Supplementary file 2 — Description of Additional Supplementary Materials [file 42003_2024_6565_MOESM2_ESM.pdf]

Description of Additional Supplementary Files

File name: Supplementary Data

Description: The source data behind the graphs

File name: Supplementary Data 2

Description: Bile acid report

File name: Supplementary Data 3

Description: Bile acid raw data
