# Supplementary material for: Nuclear factor interleukin 3 and metabolic dysfunction-associated fatty liver disease development
Source: Commun Biol. 2024 Jul 24;7:897. doi: 10.1038/s42003-024-06565-z (PMC11269659; doi:10.1038/s42003-024-06565-z)
Supplement: Supplementary file 4 — Supplementary Data 2 [file 42003_2024_6565_MOESM4_ESM.pdf]

BIOTOOLS

圖爾思生物科技股份有限公司
www.toolsbiotech.com

代謝體服務結案報告
膽酸檢測

Client Name: 陽明交通大學 吳莉玲老師

Service Number #TMNR22PBA01413

2022/08/31

BMRC

3rd Generation Sequencing

目錄

一、樣本資訊 2
二、實驗資訊 2
2.1 儀器與試劑 2
2.1.1 實驗試劑 2
2.1.2 實驗儀器 2
2.2 實驗方法 3
2.2.1 代謝物萃取 3
2.2.2 上機檢測 3
2.2.3 數據處理 4
三、檢測結果 4

一、樣本資訊

共收到客戶提供的24例小鼠糞便，實際檢測24例樣本。

表1、樣本資訊表

組別	M_WT_NCD	M_WT_HFD	M_KO_NCD	M_KO_HFD
樣本數量	3	3	3	3
實驗檢測	3	3	3	3
組別	F_WT_NCD	F_WT_HFD	F_KO_NCD	F_KO_HFD
樣本數量	3	3	3	3
實驗檢測	3	3	3	3

注：樣本收到後，立即保存在-80 °C低溫冰箱中，直至實驗檢測

二、實驗資訊

2.1 儀器與試劑

2.1.1 實驗試劑

表2. 實驗試劑列表

名稱	CAS	純度	廠牌
乙腈（Acetonitrile）	75058	LC-MS級	Honeywell
甲酸 (Formic acid)	56302	LC-MS級	Fluka

2.1.2 實驗儀器

表3. 實驗儀器清單

儀器	型號	廠牌
串聯質譜	Xevo TQS	Waters
純水機	Option-Q	ELGA
層析管柱	ACQUITY UPLC BEH C8 1.7µm 2.1*100 mm	Waters

2.2 實驗方法

2.2.1 代謝物萃取

1. 將10 mg樣本加入500 μL 萃取液 (MeoH:ACN:H_2O:Formic acid，含內標DCA-d6, GCA-d4, TCDCA-d4) ，vortex 30 s混勻；
2. 35 Hz研磨處理4 min，超音波震盪5 min（冰水浴）；
3. -20°C靜置1小時；
4. 離心12000rpm, 15分鐘，4°C； (上述步驟由客戶端完成)
5. 取上清液200 μL至進樣瓶上機檢測；

Metabolite extraction:

10mg sample were extracted with 500 μL extraction buffer containing internal standard mixture. After 30 s vortex, the samples were homogenized at 35 Hz for 4 min and sonicated for 5 min in ice-water bath. Then the samples were incubated for 1 h at -20 °C and centrifuged at 12000 rpm for 15 min at 4 °C. The supernatant were transferred to bile acid analysis. The analysis was performed on Waters ultra-high-performance liquid chromatography coupled with Waters Xevo TQS MS (Waters Corp.).

2.2.2 上機檢測

Waters超高效液相控制下按照下表中的流動相參數進行分析。所使用的色譜柱為購自Waters的UPLC BEH C8 層析管柱 (1.7 μm*2.1*100mm)，管柱控溫為60°C；進樣體積為5 μL。

使用Waters串聯質譜儀進行偵測，離子化方法為電噴霧離子法正電核模式，訊號獲取則採用多重反應監測方式，得到最佳母離子與子離子片段組合配對進行定量分析。ESI離子源參數設置如下： Capillary voltage: 1.5 KV；Desolvation gas flow rate: 1000 L/h； cone gas flow: 150 L/h；desolvation temperatures: 600°C；source temperatures: 150°C.

Mass analysis:

Chromatographic separation was performed on a Waters ACQUITY BEH C8 column (2.1 mm × 100 mm × 1.7 μm). Column temperature was maintained at 60°C. For optimized parameters, the mobile phase A was 10% acetonitrile with 0.01% formic acid and the mobile phase B was isopropanol/acetonitrile (50:50, v/v) with 0.01% formic acid.

Mass analysis was performed using the Waters Xevo TQ-S system in positive-ion ESI mode. The capillary voltage was set at 1.5 KV. Desolvation gas flow rate was set at 1000 L/h, and cone gas flow was maintained at 150 L/h. The desolvation and source temperatures were set at 600°C and 150°C, respectively. QC sample (laboratory quality control) and mix QC sample (a mixture of all samples) were prepared for analyzed during the analytical runs after every 10th sample.

2.2.3 數據處理

使用TargetLynx進行訊號強度積分與濃度換算。

三、檢測結果

共檢測到44種膽酸，詳見Excel表。

BIOTOOLS

BIOTOOLS CO., LTD.
www.toolsbiotech.com
02-26972697 02-26963689
info@toolsbiotech.com
