# Supplementary material for: Nuclear factor interleukin 3 and metabolic dysfunction-associated fatty liver disease development
Source: Commun Biol. 2024 Jul 24;7:897. doi: 10.1038/s42003-024-06565-z (PMC11269659; doi:10.1038/s42003-024-06565-z)
Supplement: Supplementary file 5 — Supplementary Data 3 NEW [file 42003_2024_6565_MOESM5_ESM.pdf]

No	Compound Name	Name	MRM	Category 1 (primary/secondary bile acids)	Category 2	CAS	Molecular formula
BA01	Ursocholanic Acid		359.2955				
BA03	5-Cholenic Acid-3 β -ol		373.2748				
BA04	3-Ketocholanic Acid; Dehydrolithocholic acid	DHLCA	373.2748	—	Free/Deconjugated	1553-56-6	C24H38O3
BA05	Lithocholic acid	LCA	375.2904	secondary bile acids	Free/Deconjugated	434-13-9	C24H40O3
BA06	Isolithocholic Acid	isoLCA	375.2904	secondary bile acids	Free/Deconjugated	1534-35-6	C24H40O3
BA09	3 α ,12 α , 23-Nordeoxycholic Acid	23norDCA	377.2697	—	Free/Deconjugated	53608-86-9	C23H38O4
BA10	9(11), (5 β)-Cholenic Acid-3 α -ol-12-one		387.2540				
BA12	3,7-Diketocholanic Acid		387.2540				
BA16	5 β -Cholenic Acid-7 α -ol-3-one		389.2697				
BA17	5 α -Cholanic Acid-3 α -ol-6-one		389.2697				
BA18	3 α -Hydroxy-7 Ketolithocholic Acid	7-ketoLCA	389.2697	secondary bile acids	Free/Deconjugated	4651-67-6	C24H38O4
BA19	3 α -Hydroxy-12 Ketolithocholic Acid	12-ketoLCA	389.2697	secondary bile acids	Free/Deconjugated	5130-29-0	C24H38O4
BA20	Murocholic Acid	MuroCA	391.2853				
BA21	Isodeoxycholic Acid	isoDCA	391.2853	—	Free/Deconjugated	566-17-6	C24H40O4
BA22	5 β -Cholanic Acid-3 β , 12 α -diol		391.2853				
BA23	Deoxycholic Acid	DCA	391.2853	secondary bile acids	Free/Deconjugated	83-44-3	C24H40O4
BA24	Chenodeoxycholic Acid	CDCA	391.2853	primary bile acids	Free/Deconjugated	474-25-9	C24H40O4
BA25	Hyodeoxycholic acid	HDCA	391.2853	secondary bile acids	Free/Deconjugated	83-49-8	C24H40O4
BA26	3,7,12 Dehydrocholic acid	DHCA	401.2333	—	Free/Deconjugated	81-23-2	C24H34O5
BA28	3 α -Hydroxy-6,7-DiketoCholanic Acid	6,7-diketoLCA	403.2489	secondary bile acids	Free/Deconjugated	-	C24H36O5
BA29	5 β -Cholanic Acid-3 α , 6 α -diol-7-one;7-Ketodeoxycholic acid	7-DHCA	405.2646	secondary bile acids	Free/Deconjugated	911-40-0	C24H38O5
BA30	3 Dehydrocholic Acid	3-DHCA	405.2646	—	Free/Deconjugated	2304-89-4	C24H38O5
BA32	Ursodeoxycholic acid	UDCA	391.2853	(Human) secondary bile acids、 (mouse) primary bile acids	Free/Deconjugated	128-13-2	C24H40O4
BA34	β Muricholic Acid	β -MCA	407.2802	primary bile acids	Free/Deconjugated	2393-59-1	C24H40O5
BA35	Cholic acid	CA	407.2802	primary bile acids	Free/Deconjugated	81-25-4	C24H40O5
BA36	Hyocholic acid (λ -MCA)	HCA	407.2802	primary bile acids	Free/Deconjugated	547-75-1	C24H40O5
BA37	α Muricholic Acid	α -MCA	407.2802	primary bile acids	Free/Deconjugated	2393-58-0	C24H40O5
BMRC20	Ursocholic acid		407.2802	—	Free/Deconjugated	2955-27-3	C24H40O5
BA39	Glycolithocholic Acid	GLCA	432.3119 → 74	secondary bile acids	conjugated	24404-83-9	C26H43NO4
BA40	Glycohyodeoxycholic Acid	GHDCA	448.3068 → 74	secondary bile acids	conjugated	38411-84-6	C26H43NO5
BA41	Glycochenodeoxycholic Acid	GCDCA	448.3068 → 74	primary bile acids	conjugated	16564-43-5	C26H43NO5
BA42	Glycodeoxycholic acid	GDCA	448.3068 → 74	secondary bile acids	conjugated	16409-34-0	C26H43NO5
BA43	Glycoursodeoxycholic Acid	GUDCA	448.3068 → 74	secondary bile acids	conjugated	64480-66-6	C26H43NO5
BA44	3,7,12 Glycodehydrocholic acid	GDHCA	458.2548 → 74	—	conjugated	3415-45-0	C26H37NO6
BA46	Glycocholic Acid	GCA	464.3017 → 74	primary bile acids	conjugated	475-31-0	C26H43NO6
BA47	Tauro-ursocholanic Acid		466.2996 → 80				
BA48	Taurolithocholic Acid	TLCA	482.2945 → 80	secondary bile acids	conjugated	6042-32-6	C26H45NO5S
BA49	Glycohyocholic Acid (Glyco- λ -muricholic acid)	GHCA (G λ MCA)	464.3017 → 74	primary bile acids	conjugated	-	C26H43NO6
BA50	Tauro-ursodeoxycholic Acid	TUDCA	498.2894 → 80	secondary bile acids	conjugated	14605-22-2	C26H45NO6S
BA51	Taurohyodeoxycholic Acid	THDCA	498.2894 → 80	secondary bile acids	conjugated	110026-03-4	C26H45NO6S
BA52	Taurochenodeoxycholic Acid	TCDCA	498.2894 → 80	primary bile acids	conjugated	516-35-8	C26H45NO6S
BA53	Taurodeoxycholic Acid	TDCA	498.2894 → 80	secondary bile acids	conjugated	1180-95-6	C26H45NO6S
BA54	3,7,12 Taurodehydrocholic acid	TDHCA	508.2374 → 80				
BA55	Taurohyocholic Acid	THCA	514.2843 → 80				
BA57	Tauro- α Muricholic Acid	T- α -MCA	514.2843 → 80	primary bile acids	conjugated	25696-60-0	C26H45NO7S
BA59	Taurocholic Acid	TCA	514.2843 → 80	primary bile acids	conjugated	81-24-3	C26H45NO7S
IS64	Deoxycholic Acid-d6		397.5886				C24D6H34O4
IS71	Glycocholic Acid-d4		468.6394 → 74			1201918-15-1	C26D4H39NO6
IS73	Taurochenodeoxycholic Acid-d4		502.7203 → 80			6009-98-9	C26H41D4NO6S
